# Supplementary material for: Maternal body composition and gestational weight gain in relation to asthma control during pregnancy
Source: PLoS One. 2022 Apr 20;17(4):e0267122. doi: 10.1371/journal.pone.0267122 (PMC9020691; doi:10.1371/journal.pone.0267122)
Supplement: S1 Table — (DOCX) [file pone.0267122.s001.docx]

| **S1 Table. Eligibility and exclusion criteria in the Breathe-Wellbeing, Environment, Lifestyle, and Lung Function Study, 2015-2019, USA.** |
| --- |
| *Eligibility criteria for all women:* |
| - Age >18 - Less than 15 weeks, 0 days gestation at time of baseline visit - English or Spanish speaking - Singleton pregnancy - Does not expect to terminate the pregnancy - Plans to deliver at the study site hospital - Willing to give blood   *Eligibility criteria for asthmatics:*   - Must have had symptoms or used prescription medication for asthma in the year prior to pregnancy   *Eligibility criteria for non-asthmatics:*   - No history of an asthma diagnosis as well as no current asthma |
| *Exclusion criteria for all women:* |
| - Diagnosis of any of the following conditions: Human Immunodeficiency Virus (HIV), Lupus, Rheumatoid Arthritis, Multiple Sclerosis, Mixed Connective Tissue Disease, cystic fibrosis |
