# Supplementary material for: Maternal body composition and gestational weight gain in relation to asthma control during pregnancy
Source: PLoS One. 2022 Apr 20;17(4):e0267122. doi: 10.1371/journal.pone.0267122 (PMC9020691; doi:10.1371/journal.pone.0267122)
Supplement: S2 Table — (DOCX) [file pone.0267122.s002.docx]

| S2 Table**.** **Definitions used to categorize women according to asthma medication regimen in the Breathe-Wellbeing, Environment, Lifestyle, and Lung Function Study, 2015-2019, USA..** | | |
| --- | --- | --- |
|  | American College of Obstetricians and Gynecologists step therapy guidelines | Our definition |
| Step 1 | No daily medication, albuterol as needed | No asthma medications or taking only reliever therapy as needed |
| Step 2 | Preferred: Low-dose inhaled corticosteroid  Alternative: Cromolyn, leukotriene receptor antagonist,  or theophylline | Taking one long-term asthma medication including an inhaled corticosteroid, cromolyn, leukotriene antagonist, or theophylline |
| Step 3 | Preferred: Low- or medium-dose inhaled corticosteroid and salmeterol  Alternative: Low- or medium- dose inhaled corticosteroid and either leukotriene receptor antagonist or theophylline | Taking at least two medications including a long-acting beta-agonist, inhaled corticosteroids, leukotriene receptor antagonist, theophylline, and/or anticholinergic |
| Step 4+ | Preferred: High-dose inhaled corticosteroid and salmeterol and (if needed) oral corticosteroid  Alternative: High-dose inhaled corticosteroid and theophylline and oral corticosteroid if needed | Taking at least three medications including a long-acting beta-agonist, inhaled corticosteroids, leukotriene receptor antagonist, theophylline, and/or anticholinergic  Any oral corticosteroid or immunomodulator |
