# Supplementary material for: Maternal body composition and gestational weight gain in relation to asthma control during pregnancy
Source: PLoS One. 2022 Apr 20;17(4):e0267122. doi: 10.1371/journal.pone.0267122 (PMC9020691; doi:10.1371/journal.pone.0267122)
Supplement: S3 Table — (DOCX) [file pone.0267122.s003.docx]

S3 Table**. Participant characteristics Among Women with Asthma by pre-pregnancy BMI in the Breathe-Wellbeing, Environment, Lifestyle, and Lung Function Study, 2015-2019, USA.**

|  | BMI < 25  (N=102) | | BMI 25 – 30  (N=48) | | BMI ≥ 30  (N=91) | | Missing BMI  (N=58) | |
| --- | --- | --- | --- | --- | --- | --- | --- | --- |
| Variable | N | % | N | % | N | % | N | % |
| Age at enrollment^a^ | 30.0 | 5.5 | 29.8 | 6.3 | 30.9 | 5.9 | 27.1 | 5.7 |
| Site |  |  |  |  |  |  |  |  |
| Northwestern University | 66 | 64.7 | 30 | 62.5 | 47 | 51.6 | 21 | 36.2 |
| University of Alabama Birmingham | 36 | 35.3 | 18 | 37.5 | 44 | 48.4 | 37 | 63.8 |
| Race/ethnicity |  |  |  |  |  |  |  |  |
| Black | 38 | 37.3 | 28 | 58.3 | 53 | 58.2 | 38 | 65.5 |
| Hispanic | 11 | 10.8 | 3 | 6.3 | 10 | 11.0 | 5 | 8.6 |
| White | 45 | 44.1 | 14 | 29.2 | 23 | 25.3 | 5 | 8.6 |
| Mixed race/other | 8 | 7.8 | 3 | 6.3 | 5 | 5.5 | 10 | 17.2 |
| Household income (quartiles) |  |  |  |  |  |  |  |  |
| < $15,000 | 20 | 19.6 | 11 | 22.9 | 27 | 29.7 | 27 | 46.6 |
| $15,000-$40,000 | 18 | 17.6 | 11 | 22.9 | 23 | 25.3 | 13 | 22.4 |
| $40,000-$120,000 | 26 | 25.5 | 14 | 29.2 | 26 | 28.6 | 11 | 19.0 |
| ≥ $120,000 | 38 | 37.3 | 12 | 25.0 | 15 | 16.5 | 7 | 12.1 |
| Marital status |  |  |  |  |  |  |  |  |
| Divorced/separated or widowed | 2 | 2.0 | 2 | 4.2 | 8 | 8.8 | 4 | 6.9 |
| Married and/or living with partner | 70 | 68.6 | 27 | 56.3 | 47 | 51.6 | 21 | 36.2 |
| Single | 30 | 29.4 | 19 | 39.6 | 36 | 39.6 | 33 | 56.9 |
| Education |  |  |  |  |  |  |  |  |
| High school or less | 17 | 16.7 | 13 | 27.1 | 38 | 41.8 | 28 | 48.3 |
| Associate's/Some college | 31 | 30.4 | 15 | 31.3 | 28 | 30.8 | 16 | 27.6 |
| Bachelor's degree | 20 | 19.6 | 10 | 20.8 | 11 | 12.1 | 6 | 10.3 |
| Master's or advanced degree | 34 | 33.3 | 10 | 20.8 | 14 | 15.4 | 8 | 13.8 |
| Parity |  |  |  |  |  |  |  |  |
| Multiparous | 55 | 53.9 | 22 | 45.8 | 54 | 59.3 | 33 | 56.9 |
| Nulliparous | 47 | 46.1 | 26 | 54.2 | 37 | 40.7 | 25 | 43.1 |
| Pre-pregnancy cigarette smoking |  |  |  |  |  |  |  |  |
| No | 74 | 72.6 | 31 | 64.6 | 56 | 18.7 | 30 | 51.7 |
| Yes | 28 | 27.5 | 17 | 35.4 | 35 | 38.5 | 27 | 46.7 |
| *Missing* | . | . | . | . | . | . | 1 | 1.7 |
| Pre-pregnancy diabetes |  |  |  |  |  |  |  |  |
| No | 99 | 97.1 | 48 | 100.0 | 82 | 90.1 | 55 | 94.8 |
| Yes | 3 | 2.9 | 0 | 0.0 | 9 | 9.9 | 3 | 5.2 |
|  |  |  |  |  |  |  |  |  |
| Pre-pregnancy hypertension |  |  |  |  |  |  |  |  |
| No | 101 | 99.0 | 42 | 87.5 | 74 | 81.3 | 52 | 89.7 |
| Yes | 1 | 1.0 | 6 | 12.5 | 17 | 18.7 | 6 | 10.3 |
| Asthma medication regimen |  |  |  |  |  |  |  |  |
| Step 1: Mild Intermittent | 59 | 57.8 | 27 | 56.3 | 41 | 45.1 | 30 | 51.7 |
| Step 2: Mild Persistent | 15 | 14.7 | 12 | 25.0 | 18 | 19.8 | 9 | 15.5 |
| Step 3: Moderate Persistent | 20 | 19.6 | 8 | 16.7 | 17 | 18.7 | 11 | 19.0 |
| Step 4: Severe Persistent | 8 | 7.8 | 1 | 2.1 | 15 | 16.5 | 8 | 13.8 |
| Baseline asthma control |  |  |  |  |  |  |  |  |
| Well-controlled | 64 | 62.7 | 22 | 45.8 | 35 | 38.5 | 24 | 41.4 |
| Poorly-controlled | 38 | 37.3 | 26 | 54.2 | 56 | 61.5 | 34 | 58.6 |

*^a^Mean and standard deviation*
