# Supplementary material for: Maternal body composition and gestational weight gain in relation to asthma control during pregnancy
Source: PLoS One. 2022 Apr 20;17(4):e0267122. doi: 10.1371/journal.pone.0267122 (PMC9020691; doi:10.1371/journal.pone.0267122)
Supplement: S4 Table — (DOCX) [file pone.0267122.s004.docx]

| S4 Table. **Adjusted^a^ association of maternal pre-pregnancy BMI and gestational weight gain with lung function in the Breathe-Wellbeing, Environment, Lifestyle, and Lung Function Study, 2015-2019, USA.** | | | | | | | | | | |
| --- | --- | --- | --- | --- | --- | --- | --- | --- | --- | --- |
|  | % FEV1 | | % FEV6 | | % FVC | | % PEF | | FEV1/FVC | |
|  | β | 95% CI | β | 95% CI | β | 95% CI | β | 95% CI | β | 95% CI |
| First trimester |  |  |  |  |  |  |  |  |  |  |
| BMI 25-30^b^ | -1.82 | -6.48, 2.85 | -4.16 | -9.71, 1.39 | -2.71 | -6.97, 1.56 | -3.45 | -9.78, 2.88 | 0.006 | -0.028, 0.040 |
| BMI ≥ 30^b^ | -3.54 | -7.63, 0.54 | -3.98 | -8.61, 0.66 | **-5.42** | **-8.97, -1.88** | -2.58 | -7.43, 2.26 | 0.003 | -0.026, 0.032 |
| First trimester GWG: inadequate^c^ | 1.15 | -7.07, 9.38 | -6.05 | -17.24, 5.14 | -1.15 | -8.71, 6.41 | 0.91 | -7.14, 8.96 | 0.014 | -0.039, 0.067 |
| First trimester GWG: excessive^c^ | 0.51 | -5.40, 6.42 | -4.00 | -11.79, 3.80 | -0.26 | -5.25, 4.73 | 1.32 | -5.09, 7.73 | -0.006 | -0.048, 0.037 |
| Second trimester |  |  |  |  |  |  |  |  |  |  |
| BMI 25-30^b^ | -1.82 | -6.31, 2.66 | -3.54 | -9.12, 2.05 | -2.65 | -6.84, 1.53 | -3.60 | -9.72, 2.52 | 0.002 | -0.034, 0.038 |
| BMI ≥ 30^b^ | -3.51 | -7.46, 0.44 | -3.85 | -8.53, 0.83 | **-5.32** | **-8.79, -1.84** | -2.98 | -7.69, 1.73 | 0.005 | -0.025, 0.036 |
| First trimester GWG: inadequate^c^ | 0.21 | -8.18, 8.60 | -4.86 | -18.09, 8.38 | -0.37 | -8.00, 7.26 | 0.02 | -8.48, 8.52 | -0.003 | -0.062, 0.056 |
| First trimester GWG: excessive^c^ | 0.27 | -6.31, 6.85 | -6.34 | -14.72, 2.04 | -2.43 | -7.81, 2.95 | -3.14 | -10.38, 4.11 | 0.014 | -0.036, 0.064 |
| Second trimester GWG: inadequate^c^ | 2.00 | -3.53, 7.53 | -0.49 | -7.79, 6.82 | -1.00 | -5.92, 3.93 | 1.48 | -5.10, 8.07 | 0.029 | -0.015, 0.073 |
| Second trimester GWG: excessive^c^ | 1.59 | -3.34, 6.53 | 4.14 | -1.51, 9.79 | 3.30 | -1.05, 7.64 | **6.98** | **0.42, 13.54** | -0.013 | -0.055, 0.030 |
| Third trimester |  |  |  |  |  |  |  |  |  |  |
| BMI 25-30^b^ | -1.87 | -6.31, 2.57 | -3.19 | -8.86, 2.48 | -2.64 | -6.75, 1.48 | -3.40 | -9.77, 2.97 | -0.001 | -0.040, 0.038 |
| BMI ≥ 30^b^ | -3.46 | -7.39, 0.46 | -3.72 | -8.48, 1.04 | **-5.20** | **-8.61, -1.78** | -3.42 | -8.42, 1.58 | 0.007 | -0.025, 0.040 |
| First trimester GWG: inadequate^c^ | -0.48 | -8.75, 7.79 | -4.83 | -18.89, 9.23 | -0.33 | -7.85, 7.19 | -1.10 | -10.60, 8.39 | -0.011 | -0.073, 0.051 |
| First trimester GWG: excessive^c^ | -0.34 | -6.90, 6.22 | -6.17 | -15.26, 2.93 | -2.01 | -7.48, 3.46 | -4.91 | -13.16, 3.34 | 0.004 | -0.049, 0.057 |
| Second trimester GWG: inadequate^c^ | 0.20 | -6.73, 7.14 | -1.05 | -10.65, 8.55 | -0.44 | -6.57, 5.69 | -2.75 | -11.37, 5.87 | 0.014 | -0.041, 0.070 |
| Second trimester GWG: excessive^c^ | 2.78 | -3.28, 8.84 | 3.62 | -3.37, 10.61 | 2.22 | -3.02, 7.45 | **8.78** | **0.62, 16.94** | 0.010 | -0.042, 0.063 |
| Third trimester GWG: inadequate^c^ | 1.69 | -3.91, 7.29 | 1.53 | -6.04, 9.10 | 0.84 | -4.30, 5.99 | 2.03 | -5.17, 9.24 | 0.004 | -0.046, 0.054 |
| Third trimester GWG: excessive^c^ | -2.08 | -8.81, 4.65 | 0.88 | -6.94, 8.69 | 2.10 | -3.41, 7.62 | -5.13 | -13.26, 3.01 | -0.036 | -0.095, 0.022 |
| *Abbreviations: % FEV1, percent predicted forced expiratory volume in 1 second; % FEV6, percent predicted forced expiratory volume in 6 seconds; % FVC, percent predicted forced vital capacity; % PEF, percent predicted peak flow; BMI, body mass index; CI, confidence interval; FEV1/FVC, ratio of forced expiratory volume in 1 second to forced vital capacity; GWG, gestational weight gain*  *Bold represents statistically significant (p ≤ 0.05) findings*  *^a^Models were adjusted for study site, age, race/ethnicity, household income, marital status, education, parity, and pre-pregnancy cigarette smoke exposure. Models for gestational weight gain were additionally adjusted for pre-pregnancy BMI, diabetes, and hypertension.*  *^b^Reference group is BMI < 25*  *^c^Reference group is adequate gestational weight gain* | | | | | | | | | | |
