# Supplementary material for: Maternal body composition and gestational weight gain in relation to asthma control during pregnancy
Source: PLoS One. 2022 Apr 20;17(4):e0267122. doi: 10.1371/journal.pone.0267122 (PMC9020691; doi:10.1371/journal.pone.0267122)
Supplement: S5 Table — (DOCX) [file pone.0267122.s005.docx]

| S5 Table. **Adjusted^a^ association between maternal pre-pregnancy BMI and gestational weight gain with incidence of asthma symptom in the Breathe-Wellbeing, Environment, Lifestyle, and Lung Function Study, 2015-2019, USA.** | | | | | | | | |
| --- | --- | --- | --- | --- | --- | --- | --- | --- |
|  | Activity limitation | | Night symptoms | | Rescue inhaler use | | Respiratory symptoms | |
|  | RR | 95% CI | RR | 95% CI | RR | 95% CI | RR | 95% CI |
| First trimester |  |  |  |  |  |  |  |  |
| BMI 25-30^b^ | 1.27 | 0.72, 2.24 | 1.41 | 0.79, 2.51 | 1.24 | 0.75, 2.04 | 1.13 | 0.81, 1.58 |
| BMI ≥ 30^b^ | 1.06 | 0.63, 1.77 | **1.66** | **1.08, 2.56** | 1.27 | 0.86, 1.88 | 1.16 | 0.90, 1.49 |
| First trimester GWG: inadequate^c^ | **3.73** | **1.18, 11.78** | 1.31 | 0.59, 2.91 | 1.92 | 0.65, 5.70 | **0.63** | **0.42, 0.95** |
| First trimester GWG: excessive^c^ | **3.36** | **1.15, 9.80** | 1.03 | 0.50, 2.13 | **2.57** | **1.01, 6.51** | 0.87 | 0.62, 1.21 |
| Second trimester |  |  |  |  |  |  |  |  |
| BMI 25-30^b^ | 1.22 | 0.70, 2.14 | 1.18 | 0.66, 2.08 | 1.27 | 0.76, 2.10 | 1.05 | 0.77, 1.43 |
| BMI ≥ 30^b^ | 0.99 | 0.65, 1.51 | 1.33 | 0.91, 1.94 | 1.34 | 0.89, 2.01 | 1.20 | 0.96, 1.50 |
| First trimester GWG: inadequate^c^ | 1.84 | 0.73, 4.63 | 0.68 | 0.33, 1.39 | 1.66 | 0.59, 4.65 | 0.69 | 0.42, 1.13 |
| First trimester GWG: excessive^c^ | **2.44** | **1.07, 5.55** | 1.09 | 0.55, 2.17 | **2.89** | **1.13, 7.41** | 1.05 | 0.72, 1.53 |
| Second trimester GWG: inadequate^c^ | 1.35 | 0.65, 2.81 | 0.94 | 0.48, 1.82 | 1.65 | 0.85, 3.20 | 0.93 | 0.67, 1.28 |
| Second trimester GWG: excessive^c^ | 1.18 | 0.56, 2.50 | 0.81 | 0.41, 1.62 | 1.35 | 0.75, 2.42 | 0.86 | 0.63, 1.20 |
| Third trimester |  |  |  |  |  |  |  |  |
| BMI 25-30^b^ | 1.27 | 0.71, 2.26 | 1.19 | 0.63, 2.26 | 1.26 | 0.75, 2.11 | 0.96 | 0.69, 1.35 |
| BMI ≥ 30^b^ | 1.14 | 0.74, 1.76 | 1.44 | 0.99, 2.11 | 1.33 | 0.88, 2.02 | 1.21 | 0.96, 1.52 |
| First trimester GWG: inadequate^c^ | 1.49 | 0.56, 3.95 | 0.67 | 0.31, 1.43 | 2.02 | 0.62, 6.56 | 0.58 | 0.33, 1.01 |
| First trimester GWG: excessive^c^ | 1.78 | 0.74, 4.23 | 1.21 | 0.57, 2.55 | **3.49** | **1.21, 10.02** | 1.05 | 0.71, 1.55 |
| Second trimester GWG: inadequate^c^ | 1.45 | 0.61, 3.45 | 0.71 | 0.31, 1.59 | 1.62 | 0.71, 3.70 | 0.94 | 0.62, 1.42 |
| Second trimester GWG: excessive^c^ | 1.76 | 0.75, 4.14 | 0.90 | 0.42, 1.94 | 1.50 | 0.73, 3.08 | 0.95 | 0.64, 1.39 |
| Third trimester GWG: inadequate^c^ | 0.66 | 0.33, 1.31 | 1.36 | 0.66, 2.77 | 0.83 | 0.45, 1.51 | 1.02 | 0.69, 1.51 |
| Third trimester GWG: excessive^c^ | 0.69 | 0.31, 1.51 | 0.86 | 0.42, 1.76 | 0.73 | 0.35, 1.52 | 0.88 | 0.60, 1.30 |
| *Abbreviations: BMI, Body mass index; CI, confidence interval; GWG, gestational weight gain; RR, relative rate ratio*  *Bold represents statistically significant (p ≤ 0.05) findings*  *^a^Models were adjusted for study site, age, race/ethnicity, household income, marital status, education, parity, and pre-pregnancy cigarette smoke exposure. Models for gestational weight gain were additionally adjusted for pre-pregnancy BMI, diabetes, and hypertension.*  *^b^Reference group is BMI < 25*  *^c^Reference group is adequate gestational weight gain* | | | | | | | | |
