# Supplementary material for: Maternal body composition and gestational weight gain in relation to asthma control during pregnancy
Source: PLoS One. 2022 Apr 20;17(4):e0267122. doi: 10.1371/journal.pone.0267122 (PMC9020691; doi:10.1371/journal.pone.0267122)
Supplement: S6 Table — (DOCX) [file pone.0267122.s006.docx]

| S6 Table. **Adjusted^a^ association between maternal body composition and gestational weight gain with individual respiratory symptoms in the Breathe-Wellbeing, Environment, Lifestyle, and Lung Function Study, 2015-2019, USA.** | | | | | | | | | | |
| --- | --- | --- | --- | --- | --- | --- | --- | --- | --- | --- |
|  | Wheeze | | Shortness of breath | | Cough | | Chest tightness | | Chest pain | |
|  | RR | 95% CI | RR | 95% CI | RR | 95% CI | RR | 95% CI | RR | 95% CI |
| First trimester |  |  |  |  |  |  |  |  |  |  |
| BMI 25-30^b^ | 0.66 | 0.33, 1.34 | 1.05 | 0.72, 1.53 | 1.29 | 0.80, 2.06 | 0.71 | 0.41, 1.24 | 0.63 | 0.30, 1.35 |
| BMI ≥ 30^b^ | 1.35 | 0.93, 1.98 | 1.09 | 0.83, 1.45 | 1.19 | 0.84, 1.68 | 0.93 | 0.64, 1.36 | 0.84 | 0.53, 1.33 |
| Subscapular skinfold^c^ | **1.24** | **1.00, 1.55** | 1.04 | 0.89, 1.20 | 1.12 | 0.94, 1.33 | 1.07 | 0.86, 1.33 | 1.14 | 0.86, 1.53 |
| Triceps skinfold^c^ | **1.36** | **1.09, 1.71** | 1.12 | 0.97, 1.31 | **1.23** | **1.02, 1.47** | 1.17 | 0.94, 1.46 | 1.26 | 0.96, 1.67 |
| Sum of skinfolds^c^ | **1.31** | **1.06, 1.62** | 1.08 | 0.94, 1.24 | 1.18 | 1.00, 1.39 | 1.12 | 0.91, 1.38 | 1.21 | 0.93, 1.58 |
| First trimester GWG: inadequate^d^ | 0.63 | 0.30, 1.36 | 0.73 | 0.47, 1.14 | 0.77 | 0.44, 1.35 | **0.40** | **0.21, 0.75** | 0.56 | 0.22, 1.42 |
| First trimester GWG: excessive^d^ | 0.95 | 0.46, 1.96 | 0.93 | 0.64, 1.36 | 0.93 | 0.59, 1.46 | **0.62** | **0.40, 0.98** | 0.92 | 0.46, 1.84 |
| Second trimester |  |  |  |  |  |  |  |  |  |  |
| BMI 25-30^b^ | 0.99 | 0.58, 1.67 | 1.18 | 0.79, 1.75 | 1.14 | 0.75, 1.73 | 0.69 | 0.41, 1.16 | 0.78 | 0.44, 1.39 |
| BMI ≥ 30^b^ | 1.27 | 0.90, 1.79 | 1.14 | 0.85, 1.52 | **1.35** | **1.01, 1.80** | 1.00 | 0.71, 1.39 | 0.81 | 0.52, 1.26 |
| Subscapular skinfold^c^ | 1.13 | 0.91, 1.40 | 1.07 | 0.91, 1.24 | 1.16 | 0.99, 1.36 | 1.11 | 0.90, 1.36 | 1.20 | 0.92, 1.56 |
| Triceps skinfold^c^ | **1.23** | **1.00, 1.51** | 1.01 | 0.85, 1.18 | 1.13 | 0.96, 1.34 | 1.20 | 0.98, 1.48 | 1.26 | 0.98, 1.62 |
| Sum of skinfolds^c^ | 1.20 | 0.98, 1.46 | 1.04 | 0.89, 1.21 | 1.15 | 0.99, 1.34 | 1.16 | 0.95, 1.40 | 1.24 | 0.97, 1.58 |
| First trimester GWG: inadequate^d^ | 0.51 | 0.23, 1.14 | 0.78 | 0.42, 1.46 | 0.82 | 0.44, 1.52 | **0.47** | **0.22, 0.99** | 0.82 | 0.35, 1.91 |
| First trimester GWG: excessive^d^ | 1.13 | 0.56, 2.26 | 0.85 | 0.53, 1.35 | 1.28 | 0.77, 2.13 | 0.97 | 0.56, 1.66 | 1.18 | 0.59, 2.36 |
| Second trimester GWG: inadequate^d^ | 1.01 | 0.56, 1.85 | 0.68 | 0.44, 1.06 | 1.04 | 0.66, 1.62 | 0.95 | 0.56, 1.60 | 0.53 | 0.27, 1.03 |
| Second trimester GWG: excessive^d^ | 0.85 | 0.50, 1.43 | 0.85 | 0.57, 1.27 | 0.87 | 0.56, 1.36 | 0.71 | 0.41, 1.25 | 0.52 | 0.28, 0.95 |
| Third trimester |  |  |  |  |  |  |  |  |  |  |
| BMI 25-30^b^ | 1.01 | 0.59, 1.74 | 1.12 | 0.75, 1.67 | 1.13 | 0.73, 1.75 | 0.57 | 0.31, 1.04 | 0.60 | 0.32, 1.11 |
| BMI ≥ 30^b^ | 1.33 | 0.93, 1.88 | 1.14 | 0.85, 1.52 | **1.46** | **1.09, 1.94** | 0.98 | 0.69, 1.39 | **0.60** | **0.38, 0.94** |
| Subscapular skinfold^c^ | 1.15 | 0.92, 1.43 | 1.05 | 0.89, 1.23 | **1.18** | **1.00, 1.38** | 1.03 | 0.82, 1.28 | 1.03 | 0.78, 1.36 |
| Triceps skinfold^c^ | 1.22 | 0.99, 1.51 | 0.99 | 0.84, 1.17 | 1.15 | 0.97, 1.36 | 1.18 | 0.94, 1.48 | 1.17 | 0.90, 1.52 |
| Sum of skinfolds^c^ | 1.20 | 0.98, 1.48 | 1.02 | 0.87, 1.19 | 1.17 | 1.00, 1.37 | 1.10 | 0.89, 1.36 | 1.11 | 0.86, 1.43 |
| First trimester GWG: inadequate^d^ | 0.41 | 0.17, 0.98 | 0.69 | 0.34, 1.41 | 0.71 | 0.36, 1.41 | **0.39** | **0.17, 0.93** | 0.95 | 0.38, 2.39 |
| First trimester GWG: excessive^d^ | 1.20 | 0.59, 2.42 | 0.85 | 0.52, 1.37 | 1.21 | 0.71, 2.09 | 1.21 | 0.70, 2.11 | 1.55 | 0.75, 3.21 |
| Second trimester GWG: inadequate^d^ | 0.92 | 0.44, 1.95 | 0.69 | 0.41, 1.18 | 1.04 | 0.61, 1.78 | 0.82 | 0.43, 1.56 | **0.38** | **0.17, 0.89** |
| Second trimester GWG: excessive^d^ | 0.92 | 0.50, 1.69 | 0.84 | 0.51, 1.38 | 1.03 | 0.60, 1.77 | 0.83 | 0.40, 1.73 | 0.71 | 0.32, 1.57 |
| Third trimester GWG: inadequate^d^ | 1.45 | 0.75, 2.81 | 0.89 | 0.56, 1.41 | 0.78 | 0.47, 1.29 | 1.38 | 0.78, 2.46 | 1.57 | 0.77, 3.20 |
| Third trimester GWG: excessive^d^ | 1.08 | 0.58, 2.02 | 0.87 | 0.53, 1.42 | 0.73 | 0.45, 1.18 | 0.74 | 0.38, 1.45 | 0.57 | 0.26, 1.25 |
| *Abbreviations: BMI, Body mass index; CI, confidence interval; GWG, gestational weight gain; RR, relative rate ratio*  *Bold represents statistically significant (p ≤ 0.05) findings*  *^a^Models were adjusted for study site, age, race/ethnicity, household income, marital status, education, parity, and pre-pregnancy cigarette smoke exposure. Models for gestational weight gain were additionally adjusted for pre-pregnancy BMI, diabetes, and hypertension.*  *^b^Reference group is BMI < 25*  *^c^For a 1-interquartile range increase, which is 13 millimeters for subscapular and triceps skinfolds, and 22.5 milimeters for the sum of skinfolds*  *^d^Reference group is adequate gestational weight gain* | | | | | | | | | | |
