# Supplementary material for: Maternal body composition and gestational weight gain in relation to asthma control during pregnancy
Source: PLoS One. 2022 Apr 20;17(4):e0267122. doi: 10.1371/journal.pone.0267122 (PMC9020691; doi:10.1371/journal.pone.0267122)
Supplement: S7 Table — (DOCX) [file pone.0267122.s007.docx]

| S7 Table. **Adjusted^a^ association of maternal body composition and gestational weight gain with asthma exacerbations across pregnancy in the Breathe-Wellbeing, Environment, Lifestyle, and Lung Function Study, 2015-2019, USA.** | | | | | | | | | | |
| --- | --- | --- | --- | --- | --- | --- | --- | --- | --- | --- |
|  | Asthma attacks | | Medical encounters | | Hospitalization*^,e^* | | Emergency department/Urgent care visit | | Doctor’s visit | |
|  | RR | 95% CI | RR | 95% CI | RR | 95% CI | RR | 95% CI | RR | 95% CI |
| BMI 25-30^b^ | 0.60 | 0.33, 1.12 | **0.41** | **0.21, 0.82** | - | - | 1.38 | 0.64, 2.96 | **0.18** | **0.06, 0.53** |
| BMI ≥ 30^b^ | 0.80 | 0.52, 1.24 | 0.75 | 0.48, 1.18 | - | - | 1.46 | 0.82, 2.63 | **0.47** | **0.28, 0.79** |
| Subscapular skinfold^c^ | 0.81 | 0.61, 1.08 | 0.94 | 0.72, 1.22 | - | - | **1.43** | **1.05, 1.94** | **0.71** | **0.52, 0.97** |
| Triceps skinfold^c^ | 0.82 | 0.62, 1.09 | 0.89 | 0.68, 1.18 | - | - | 1.04 | 0.74, 1.47 | 0.74 | 0.53, 1.01 |
| Sum of skinfolds^c^ | 0.81 | 0.62, 1.06 | 0.91 | 0.71, 1.18 | - | - | 1.25 | 0.92, 1.69 | **0.72** | **0.53, 0.97** |
| First trimester GWG: inadequate^d^ | 0.47 | 0.21, 1.04 | 1.05 | 0.32, 3.44 | - | - | 0.46 | 0.10, 2.09 | 1.76 | 0.31, 9.91 |
| First trimester GWG: excessive^d^ | 0.69 | 0.35, 1.34 | 1.87 | 0.76, 4.59 | - | - | 1.11 | 0.39, 3.20 | 3.28 | 0.94, 11.44 |
| Second trimester GWG: inadequate^d^ | 0.46 | 0.15, 1.42 | 0.82 | 0.32, 2.08 | - | - | 1.33 | 0.36, 4.88 | 0.95 | 0.33, 2.73 |
| Second trimester GWG: excessive^d^ | 0.92 | 0.47, 1.79 | 0.58 | 0.27, 1.22 | - | - | 0.38 | 0.14, 1.02 | 1.06 | 0.37, 3.02 |
| Third trimester GWG: inadequate^d^ | 1.84 | 0.81, 4.16 | 1.15 | 0.55, 2.38 | - | - | 0.67 | 0.23, 1.99 | 1.24 | 0.54, 2.88 |
| Third trimester GWG: excessive^d^ | 1.34 | 0.61, 2.96 | 1.41 | 0.68, 2.94 | - | - | **3.08** | **1.12, 8.48** | 0.80 | 0.28, 2.23 |
| *Abbreviations: BMI, Body mass index; CI, confidence interval; GWG, gestational weight gain*  *Bold represents statistically significant (p ≤ 0.05) findings*  *^a^Models were adjusted for study site, age, race/ethnicity, household income, marital status, education, parity, and pre-pregnancy cigarette smoke exposure. Models for gestational weight gain were additionally adjusted for pre-pregnancy BMI, diabetes, and hypertension.*  *^b^Reference group is BMI < 25*  *^c^For a 1-IQR increase. For subscapular and triceps skinfolds, the IQR is 13.0 millimeters. For the sum of skinfolds, the IQR is 22.5 milimeters.*  *^d^Reference group is adequate gestational weight gain*  *^e^Models failed to converge due to insufficient outcome incidence* | | | | | | | | | | |
