# Supplementary material for: Maternal body composition and gestational weight gain in relation to asthma control during pregnancy
Source: PLoS One. 2022 Apr 20;17(4):e0267122. doi: 10.1371/journal.pone.0267122 (PMC9020691; doi:10.1371/journal.pone.0267122)
Supplement: S8 Table — (DOCX) [file pone.0267122.s008.docx]

| S8 Table. **Sensitivity analyses for adjusted^a^ association of maternal pre-pregnancy BMI and gestational weight gain with lung function in the Breathe-Wellbeing, Environment, Lifestyle, and Lung Function Study, 2015-2019, USA.** | | | | | | | | | | |
| --- | --- | --- | --- | --- | --- | --- | --- | --- | --- | --- |
|  | % FEV1 | | % FEV6 | | % FVC | | % PEF | | FEV1/FVC | |
|  | β | 95% CI | β | 95% CI | β | 95% CI | β | 95% CI | β | 95% CI |
| First trimester |  |  |  |  |  |  |  |  |  |  |
| BMI 25-30^b^ | -1.54 | -6.26, 3.17 | -3.37 | -8.90, 2.15 | -2.13 | -6.39, 2.14 | -3.28 | -9.54, 2.98 | 0.005 | -0.029, 0.039 |
| BMI ≥ 30^b^ | -3.47 | -7.63, 0.68 | -3.71 | -8.35, 0.93 | **-5.03** | **-8.59, -1.46** | -2.98 | -7.89, 1.93 | 0.001 | -0.028, 0.030 |
| Subscapular skinfold^c^ | **-3.56** | **-5.84, -1.28** | **-3.39** | **-5.88, -0.91** | **-3.97** | **-5.95, -2.00** | 0.31 | -2.43, 3.06 | -0.003 | -0.020, 0.013 |
| Triceps skinfold^c^ | -2.18 | -4.56, 0.20 | -2.61 | -5.33, 0.11 | **-3.49** | **-5.54, -1.43** | -0.48 | -3.31, 2.35 | 0.008 | -0.009, 0.025 |
| Sum of skinfolds^c^ | **-3.00** | **-5.20, -0.80** | **-3.08** | **-5.52, -0.65** | **-3.88** | **-5.78, -1.98** | -0.07 | -2.72, 2.57 | 0.002 | -0.014, 0.018 |
| First trimester GWG: inadequate^d^ | 1.21 | -7.12, 9.53 | -6.13 | -17.44, 5.18 | -1.17 | -8.82, 6.48 | 1.24 | -6.82, 9.30 | 0.016 | -0.038, 0.070 |
| First trimester GWG: excessive^d^ | 0.44 | -5.50, 6.38 | -4.24 | -12.18, 3.71 | -0.43 | -5.44, 4.59 | 1.15 | -5.29, 7.60 | -0.007 | -0.050, 0.036 |
| Second trimester |  |  |  |  |  |  |  |  |  |  |
| BMI 25-30^b^ | -1.50 | -6.03, 3.03 | -2.75 | -8.30, 2.81 | -2.08 | -6.26, 2.10 | -3.25 | -9.31, 2.82 | 0.002 | -0.035, 0.038 |
| BMI ≥ 30^b^ | -3.37 | -7.39, 0.65 | -3.61 | -8.28, 1.06 | **-4.93** | **-8.42, -1.43** | -3.22 | -7.99, 1.56 | 0.004 | -0.026, 0.035 |
| Subscapular skinfold^c^ | **-3.42** | **-5.61, -1.23** | **-3.39** | **-5.89, -0.88** | **-3.89** | **-5.83, -1.96** | 0.09 | -2.58, 2.77 | -0.002 | -0.019, 0.015 |
| Triceps skinfold^c^ | **-2.29** | **-4.57, -0.00** | -2.49 | -5.24, 0.25 | **-3.42** | **-5.43, -1.41** | -0.77 | -3.53, 1.98 | 0.006 | -0.012, 0.024 |
| Sum of skinfolds^c^ | **-2.98** | **-5.10, -0.86** | **-3.03** | **-5.48, -0.58** | **-3.80** | **-5.66, -1.94** | -0.34 | -2.91, 2.23 | 0.002 | -0.014, 0.019 |
| First trimester GWG: inadequate^d^ | 0.25 | -8.22, 8.72 | -4.72 | -17.90, 8.45 | -0.29 | -7.94, 7.37 | 0.34 | -8.09, 8.76 | -0.002 | -0.061, 0.058 |
| First trimester GWG: excessive^d^ | 0.18 | -6.45, 6.82 | -6.63 | -15.20, 1.95 | -2.65 | -8.06, 2.76 | -3.42 | -10.74, 3.89 | 0.013 | -0.037, 0.063 |
| Second trimester GWG: inadequate^d^ | 1.95 | -3.64, 7.54 | -0.74 | -7.81, 6.33 | -1.34 | -6.23, 3.55 | 1.73 | -4.85, 8.32 | 0.030 | -0.015, 0.075 |
| Second trimester GWG: excessive^d^ | 1.60 | -3.38, 6.58 | 3.92 | -1.63, 9.47 | 3.09 | -1.20, 7.38 | **7.35** | **0.85, 13.85** | -0.012 | -0.055, 0.031 |
| Third trimester |  |  |  |  |  |  |  |  |  |  |
| BMI 25-30^b^ | -1.51 | -5.99, 2.97 | -2.38 | -8.02, 3.26 | -2.08 | -6.19, 2.03 | -2.91 | -9.22, 3.40 | -0.001 | -0.040, 0.039 |
| BMI ≥ 30^b^ | -3.27 | -7.26, 0.71 | -3.49 | -8.24, 1.26 | **-4.82** | **-8.25, -1.39** | -3.56 | -8.62, 1.51 | 0.007 | -0.026, 0.040 |
| Subscapular skinfold^c^ | **-3.32** | **-5.49, -1.15** | **-3.37** | **-5.92, -0.82** | **-3.79** | **-5.69, -1.89** | -0.31 | -3.14, 2.53 | 0.000 | -0.019, 0.018 |
| Triceps skinfold^c^ | **-2.37** | **-4.63, -0.12** | -2.38 | -5.17, 0.42 | **-3.32** | **-5.30, -1.35** | -1.20 | -4.11, 1.72 | 0.006 | -0.013, 0.025 |
| Sum of skinfolds^c^ | **-2.97** | **-5.06, -0.88** | **-2.96** | **-5.46, -0.47** | **-3.70** | **-5.53, -1.87** | -0.77 | -3.49, 1.96 | 0.003 | -0.015, 0.020 |
| First trimester GWG: inadequate^d^ | -0.46 | -8.81, 7.90 | -4.71 | -18.71, 9.28 | -0.25 | -7.79, 7.29 | -0.77 | -10.10, 8.56 | -0.011 | -0.074, 0.052 |
| First trimester GWG: excessive^d^ | -0.32 | -6.96, 6.32 | -6.02 | -15.33, 3.29 | -1.99 | -7.49, 3.51 | -5.23 | -13.55, 3.09 | 0.003 | -0.051, 0.057 |
| Second trimester GWG: inadequate^d^ | 0.16 | -6.80, 7.13 | -1.50 | -10.77, 7.77 | -0.90 | -6.97, 5.17 | -2.33 | -10.95, 6.29 | 0.016 | -0.041, 0.073 |
| Second trimester GWG: excessive^d^ | 2.59 | -3.55, 8.73 | 2.45 | -4.46, 9.36 | 1.60 | -3.52, 6.73 | **9.09** | **1.02, 17.15** | 0.012 | -0.041, 0.065 |
| Third trimester GWG: inadequate^d^ | 1.90 | -3.81, 7.61 | 3.49 | -4.12, 11.09 | 1.70 | -3.42, 6.82 | 1.61 | -5.81, 9.03 | 0.002 | -0.049, 0.054 |
| Third trimester GWG: excessive^d^ | -1.75 | -8.58, 5.09 | 2.90 | -4.85, 10.64 | 2.93 | -2.49, 8.36 | -5.12 | -13.26, 3.01 | -0.038 | -0.097, 0.022 |
| *Abbreviations: % FEV1, percent predicted forced expiratory volume in 1 second; % FEV6, percent predicted forced expiratory volume in 6 seconds; % FVC, percent predicted forced vital capacity; % PEF, percent predicted peak flow; BMI, body mass index; CI, confidence interval; FEV1/FVC, ratio of forced expiratory volume in 1 second to forced vital capacity; GWG, gestational weight gain*  *Bold represents statistically significant (p ≤ 0.05) findings*  *^a^Models were adjusted for study site, age, race/ethnicity, household income, marital status, education, parity, pre-pregnancy cigarette smoke exposure, baseline asthma medication regimen, and baseline asthma control. Models for gestational weight gain were additionally adjusted for pre-pregnancy BMI, diabetes, and hypertension.*  *^b^Reference group is BMI < 25*  *^c^For a 1-IQR increase. For subscapular and triceps skinfolds, the IQR is 13.0 millimeters. For the sum of skinfolds, the IQR is 22.5 milimeters.*  *^d^Reference group is adequate gestational weight gain* | | | | | | | | | | |
