# Supplementary material for: Maternal body composition and gestational weight gain in relation to asthma control during pregnancy
Source: PLoS One. 2022 Apr 20;17(4):e0267122. doi: 10.1371/journal.pone.0267122 (PMC9020691; doi:10.1371/journal.pone.0267122)
Supplement: S9 Table — (DOCX) [file pone.0267122.s009.docx]

| S9 Table. **Sensitivity analyses for adjusted^a^ association between maternal pre-pregnancy BMI and gestational weight gain with incidence of asthma symptom in the Breathe-Wellbeing, Environment, Lifestyle, and Lung Function Study, 2015-2019, USA.** | | | | | | | | |
| --- | --- | --- | --- | --- | --- | --- | --- | --- |
|  | Activity limitation | | Night symptoms | | Rescue inhaler use | | Respiratory symptoms | |
|  | RR | 95% CI | RR | 95% CI | RR | 95% CI | RR | 95% CI |
| First trimester |  |  |  |  |  |  |  |  |
| BMI 25-30^b^ | 1.15 | 0.65, 2.04 | 1.18 | 0.71, 1.95 | 1.26 | 0.75, 2.13 | 1.08 | 0.80, 1.47 |
| BMI ≥ 30^b^ | 0.96 | 0.56, 1.62 | 1.36 | 0.91, 2.03 | 1.14 | 0.76, 1.71 | 1.10 | 0.86, 1.40 |
| Subscapular skinfold^c^ | 1.06 | 0.81, 1.39 | 1.07 | 0.86, 1.34 | 0.92 | 0.74, 1.15 | 1.02 | 0.89, 1.16 |
| Triceps skinfold^c^ | **1.57** | **1.21, 2.05** | 1.22 | 0.97, 1.54 | 1.20 | 0.96, 1.50 | 1.05 | 0.91, 1.22 |
| Sum of skinfolds^c^ | **1.29** | **1.01, 1.65** | 1.15 | 0.93, 1.43 | 1.05 | 0.85, 1.30 | 1.03 | 0.91, 1.18 |
| First trimester GWG: inadequate^d^ | **4.16** | **1.29, 13.34** | 1.58 | 0.75, 3.33 | 2.11 | 0.75, 5.94 | **0.67** | **0.45, 1.00** |
| First trimester GWG: excessive^d^ | **3.36** | **1.15, 9.81** | 0.99 | 0.51, 1.92 | **2.57** | **1.04, 6.35** | 0.86 | 0.62, 1.18 |
| Second trimester |  |  |  |  |  |  |  |  |
| BMI 25-30^b^ | 1.15 | 0.67, 1.98 | 1.07 | 0.63, 1.81 | 1.34 | 0.79, 2.28 | 1.03 | 0.76, 1.40 |
| BMI ≥ 30^b^ | 0.91 | 0.60, 1.38 | 1.10 | 0.78, 1.55 | 1.26 | 0.85, 1.87 | 1.14 | 0.92, 1.42 |
| Subscapular skinfold^c^ | 1.00 | 0.81, 1.24 | 1.08 | 0.88, 1.31 | 0.97 | 0.78, 1.21 | 1.05 | 0.93, 1.19 |
| Triceps skinfold^c^ | 1.07 | 0.85, 1.36 | 1.04 | 0.86, 1.27 | 1.23 | 0.98, 1.54 | 1.04 | 0.91, 1.18 |
| Sum of skinfolds^c^ | 1.03 | 0.84, 1.28 | 1.06 | 0.88, 1.29 | 1.09 | 0.88, 1.36 | 1.05 | 0.93, 1.18 |
| First trimester GWG: inadequate^d^ | 1.92 | 0.82, 4.48 | 0.73 | 0.39, 1.38 | 1.79 | 0.66, 4.85 | 0.72 | 0.46, 1.13 |
| First trimester GWG: excessive^d^ | **2.20** | **1.03, 4.73** | 0.94 | 0.50, 1.75 | 2.50 | 0.97, 6.41 | 1.00 | 0.70, 1.42 |
| Second trimester GWG: inadequate^d^ | 1.39 | 0.71, 2.74 | 1.05 | 0.59, 1.88 | 1.49 | 0.78, 2.83 | 0.95 | 0.70, 1.31 |
| Second trimester GWG: excessive^d^ | 1.21 | 0.56, 2.60 | 0.92 | 0.50, 1.72 | 1.34 | 0.75, 2.39 | 0.90 | 0.66, 1.24 |
| Third trimester |  |  |  |  |  |  |  |  |
| BMI 25-30^b^ | 1.23 | 0.71, 2.13 | 1.12 | 0.60, 2.08 | 1.34 | 0.78, 2.29 | 0.97 | 0.70, 1.35 |
| BMI ≥ 30^b^ | 1.09 | 0.72, 1.66 | 1.19 | 0.84, 1.69 | 1.21 | 0.81, 1.82 | 1.16 | 0.92, 1.45 |
| Subscapular skinfold^c^ | 1.11 | 0.90, 1.37 | 1.12 | 0.92, 1.37 | 0.97 | 0.77, 1.22 | 1.03 | 0.91, 1.17 |
| Triceps skinfold^c^ | 1.13 | 0.89, 1.45 | 1.10 | 0.91, 1.34 | 1.22 | 0.97, 1.53 | 1.03 | 0.90, 1.18 |
| Sum of skinfolds^c^ | 1.12 | 0.91, 1.39 | 1.12 | 0.93, 1.36 | 1.09 | 0.87, 1.37 | 1.03 | 0.91, 1.17 |
| First trimester GWG: inadequate^d^ | 1.60 | 0.64, 4.01 | 0.71 | 0.36, 1.41 | 2.29 | 0.72, 7.29 | **0.60** | **0.36, 1.00** |
| First trimester GWG: excessive^d^ | 1.63 | 0.73, 3.61 | 0.98 | 0.49, 1.97 | 3.03 | 1.03, 8.89 | 0.94 | 0.65, 1.36 |
| Second trimester GWG: inadequate^d^ | 1.59 | 0.69, 3.66 | 0.91 | 0.39, 2.14 | 1.66 | 0.75, 3.68 | 0.99 | 0.66, 1.50 |
| Second trimester GWG: excessive^d^ | 1.87 | 0.80, 4.40 | 0.99 | 0.51, 1.91 | 1.37 | 0.66, 2.87 | 1.00 | 0.70, 1.44 |
| Third trimester GWG: inadequate^d^ | 0.63 | 0.31, 1.28 | 1.07 | 0.53, 2.12 | 0.66 | 0.37, 1.21 | 0.92 | 0.62, 1.36 |
| Third trimester GWG: excessive^d^ | 0.70 | 0.32, 1.51 | 0.88 | 0.46, 1.71 | 0.78 | 0.39, 1.55 | 0.85 | 0.58, 1.23 |
| *Abbreviations: BMI, Body mass index; CI, confidence interval; GWG, gestational weight gain; RR, relative rate ratio*  *Bold represents statistically significant (p ≤ 0.05) findings*  *^a^Models were adjusted for study site, age, race/ethnicity, household income, marital status, education, parity, pre-pregnancy cigarette smoke exposure, baseline asthma medication regimen, and baseline asthma control. Models for gestational weight gain were additionally adjusted for pre-pregnancy BMI, diabetes, and hypertension.*  *^b^Reference group is BMI < 25*  *^c^For a 1-IQR increase. For subscapular and triceps skinfolds, the IQR is 13.0 millimeters. For the sum of skinfolds, the IQR is 22.5 milimeters.*  *^d^Reference group is adequate gestational weight gain* | | | | | | | | |
