# Supplementary material for: Maternal body composition and gestational weight gain in relation to asthma control during pregnancy
Source: PLoS One. 2022 Apr 20;17(4):e0267122. doi: 10.1371/journal.pone.0267122 (PMC9020691; doi:10.1371/journal.pone.0267122)
Supplement: S10 Table — (DOCX) [file pone.0267122.s010.docx]

| S10 Table. **Sensitivity analyses for adjusted^a^ association of maternal body composition and gestational weight gain with asthma exacerbations across pregnancy in the Breathe-Wellbeing, Environment, Lifestyle, and Lung Function Study, 2015-2019, USA.** | | | | |
| --- | --- | --- | --- | --- |
|  | Asthma attacks | | Medical encounters | |
|  | RR | 95% CI | RR | 95% CI |
| BMI 25-30^b^ | 0.55 | 0.29, 1.05 | **0.36** | **0.19, 0.69** |
| BMI ≥ 30^b^ | 0.69 | 0.45, 1.07 | **0.63** | **0.41, 0.96** |
| Subscapular skinfold^c^ | 0.78 | 0.59, 1.03 | 0.84 | 0.66, 1.08 |
| Triceps skinfold^c^ | 0.78 | 0.60, 1.03 | 0.78 | 0.61, 1.01 |
| Sum of skinfolds^c^ | 0.77 | 0.60, 1.01 | 0.81 | 0.64, 1.02 |
| First trimester GWG: inadequate^d^ | 0.55 | 0.25, 1.22 | 1.60 | 0.57, 4.53 |
| First trimester GWG: excessive^d^ | 0.55 | 0.29, 1.03 | 1.47 | 0.59, 3.64 |
| Second trimester GWG: inadequate^d^ | 0.52 | 0.16, 1.67 | 1.03 | 0.39, 2.70 |
| Second trimester GWG: excessive^d^ | 1.20 | 0.64, 2.27 | 0.66 | 0.32, 1.35 |
| Third trimester GWG: inadequate^d^ | 1.51 | 0.63, 3.62 | 0.73 | 0.33, 1.59 |
| Third trimester GWG: excessive^d^ | 1.13 | 0.53, 2.39 | 1.32 | 0.64, 2.74 |
| *Abbreviations: BMI, Body mass index; CI, confidence interval; GWG, gestational weight gain*  *Bold represents statistically significant (p ≤ 0.05) findings*  *^a^Models were adjusted for study site, age, race/ethnicity, household income, marital status, education, parity, pre-pregnancy cigarette smoke exposure, baseline asthma medication regimen, and baseline asthma control. Models for gestational weight gain were additionally adjusted for pre-pregnancy BMI, diabetes, and hypertension.*  *^b^Reference group is BMI < 25*  *^c^For a 1-IQR increase. For subscapular and triceps skinfolds, the IQR is 13.0 millimeters. For the sum of skinfolds, the IQR is 22.5 milimeters.*  *^d^Reference group is adequate gestational weight gain* | | | | |
