# Supplementary material for: Maternal body composition and gestational weight gain in relation to asthma control during pregnancy
Source: PLoS One. 2022 Apr 20;17(4):e0267122. doi: 10.1371/journal.pone.0267122 (PMC9020691; doi:10.1371/journal.pone.0267122)
Supplement: S11 Table — (DOCX) [file pone.0267122.s011.docx]

| S11 Table. **Sensitivity analyses for adjusted^a^ association of maternal pre-pregnancy BMI and gestational weight gain with lung function among women without asthma in the Breathe-Wellbeing, Environment, Lifestyle, and Lung Function Study, 2015-2019, USA.** | | | | | | | | | | |
| --- | --- | --- | --- | --- | --- | --- | --- | --- | --- | --- |
|  | % FEV1 | | % FEV6 | | % FVC | | % PEF | | FEV1/FVC | |
|  | β | 95% CI | β | 95% CI | β | 95% CI | β | 95% CI | β | 95% CI |
| First trimester |  |  |  |  |  |  |  |  |  |  |
| BMI 25-30^b^ | -0.10 | -7.89, 7.69 | 3.01 | -6.90, 12.93 | 0.62 | -5.82, 7.05 | 2.94 | -7.96, 13.85 | -0.001 | -0.054, 0.051 |
| BMI ≥ 30^b^ | -4.38 | -11.95, 3.19 | -4.34 | -13.75, 5.08 | -3.70 | -10.19, 2.79 | **-11.20** | **-22.03, -0.38** | -0.006 | -0.055, 0.044 |
| Subscapular skinfold^c^ | -3.09 | -8.88, 2.70 | **-8.09** | **-15.10, -1.09** | -3.79 | -8.70, 1.11 | -2.67 | -11.29, 5.95 | -0.007 | -0.045, 0.031 |
| Triceps skinfold^c^ | 0.31 | -3.83, 4.44 | -1.46 | -7.63, 4.71 | -0.45 | -3.98, 3.07 | -2.44 | -8.55, 3.67 | 0.007 | -0.020, 0.034 |
| Sum of skinfolds^c^ | -0.97 | -5.77, 3.82 | -4.63 | -11.03, 1.77 | -1.84 | -5.92, 2.24 | -2.95 | -10.04, 4.14 | 0.003 | -0.028, 0.034 |
| First trimester GWG: inadequate^d^ | 0.66 | -10.10, 11.42 | 6.33 | -8.13, 20.80 | 2.44 | -7.11, 11.99 | -6.96 | -22.24, 8.33 | 0.007 | -0.066, 0.081 |
| First trimester GWG: excessive^d^ | 1.45 | -7.45, 10.35 | -1.06 | -13.94, 11.82 | -0.56 | -8.17, 7.05 | **-13.19** | **-25.78, -0.60** | 0.026 | -0.036, 0.087 |
| Second trimester |  |  |  |  |  |  |  |  |  |  |
| BMI 25-30^b^ | -0.10 | -7.91, 7.71 | 2.91 | -6.64, 12.46 | 0.95 | -5.30, 7.20 | 2.71 | -8.14, 13.57 | -0.001 | -0.058, 0.055 |
| BMI ≥ 30^b^ | -4.39 | -11.98, 3.20 | -4.18 | -13.25, 4.89 | -4.32 | -10.64, 2.01 | -10.22 | -20.97, 0.52 | -0.006 | -0.059, 0.047 |
| Subscapular skinfold^c^ | -3.10 | -8.90, 2.70 | **-7.80** | **-14.54, -1.05** | -4.73 | -9.50, 0.04 | -2.95 | -11.44, 5.53 | -0.007 | -0.048, 0.034 |
| Triceps skinfold^c^ | 0.31 | -3.84, 4.46 | -1.41 | -7.35, 4.53 | -0.70 | -4.16, 2.75 | -3.70 | -9.69, 2.29 | 0.008 | -0.021, 0.037 |
| Sum of skinfolds^c^ | -0.98 | -5.79, 3.83 | -4.46 | -10.62, 1.70 | -2.40 | -6.39, 1.58 | -4.04 | -11.00, 2.92 | 0.003 | -0.030, 0.037 |
| First trimester GWG: inadequate^d^ | -0.20 | -10.93, 10.53 | 4.71 | -8.84, 18.27 | 1.89 | -7.40, 11.18 | -10.07 | -25.98, 5.83 | 0.006 | -0.074, 0.086 |
| First trimester GWG: excessive^d^ | 4.88 | -5.27, 15.03 | 2.51 | -11.51, 16.54 | 2.20 | -6.08, 10.47 | -10.25 | -25.18, 4.68 | 0.036 | -0.040, 0.113 |
| Second trimester GWG: inadequate^d^ | -0.85 | -14.00, 12.31 | -1.66 | -17.77, 14.45 | -1.11 | -11.47, 9.26 | 1.37 | -17.58, 20.33 | -0.002 | -0.095, 0.091 |
| Second trimester GWG: excessive^d^ | -8.68 | -20.65, 3.29 | -10.97 | -25.80, 3.86 | -8.92 | -18.82, 0.99 | -2.90 | -20.96, 15.16 | -0.021 | -0.107, 0.064 |
| Third trimester |  |  |  |  |  |  |  |  |  |  |
| BMI 25-30^b^ | -0.05 | -7.90, 7.79 | 2.75 | -6.54, 12.04 | 1.21 | -5.26, 7.68 | 2.35 | -9.33, 14.02 | -0.002 | -0.061, 0.057 |
| BMI ≥ 30^b^ | -4.41 | -12.04, 3.22 | -4.09 | -12.90, 4.73 | -4.41 | -10.97, 2.15 | -9.68 | -21.22, 1.85 | -0.006 | -0.062, 0.050 |
| Subscapular skinfold^c^ | -3.11 | -8.94, 2.72 | **-7.62** | **-14.17, -1.07** | **-5.20** | **-10.14, -0.27** | -3.27 | -12.29, 5.74 | -0.007 | -0.050, 0.036 |
| Triceps skinfold^c^ | 0.30 | -3.87, 4.46 | -1.37 | -7.15, 4.40 | -0.80 | -4.39, 2.79 | -4.57 | -10.91, 1.78 | 0.008 | -0.022, 0.039 |
| Sum of skinfolds^c^ | -0.99 | -5.82, 3.84 | -4.36 | -10.34, 1.63 | -2.67 | -6.80, 1.47 | -4.84 | -12.23, 2.54 | 0.004 | -0.032, 0.039 |
| First trimester GWG: inadequate^d^ | -0.01 | -10.82, 10.79 | 5.46 | -7.66, 18.59 | 2.39 | -7.35, 12.13 | -11.46 | -29.35, 6.43 | 0.009 | -0.075, 0.093 |
| First trimester GWG: excessive^d^ | 4.95 | -5.33, 15.22 | 4.68 | -9.22, 18.58 | 1.92 | -6.67, 10.51 | -10.04 | -26.64, 6.57 | 0.039 | -0.041, 0.119 |
| Second trimester GWG: inadequate^d^ | -1.62 | -17.09, 13.86 | -7.13 | -24.20, 9.95 | -4.15 | -16.81, 8.50 | -3.80 | -29.50, 21.90 | 0.009 | -0.107, 0.125 |
| Second trimester GWG: excessive^d^ | -6.80 | -20.96, 7.36 | -5.11 | -21.41, 11.19 | -7.68 | -19.36, 4.00 | -0.62 | -22.45, 21.21 | -0.020 | -0.126, 0.086 |
| Third trimester GWG: inadequate^d^ | -1.73 | -12.06, 8.60 | 8.91 | -2.49, 20.30 | -1.27 | -9.73, 7.19 | -0.05 | -17.36, 17.26 | -0.023 | -0.102, 0.055 |
| Third trimester GWG: excessive^d^ | -4.61 | -19.05, 9.82 | -5.54 | -19.52, 8.45 | -6.42 | -17.61, 4.77 | -7.60 | -28.90, 13.69 | -0.011 | -0.125, 0.104 |
| *Abbreviations: % FEV1, percent predicted forced expiratory volume in 1 second; % FEV6, percent predicted forced expiratory volume in 6 seconds; % FVC, percent predicted forced vital capacity; % PEF, percent predicted peak flow; BMI, body mass index; CI, confidence interval; FEV1/FVC, ratio of forced expiratory volume in 1 second to forced vital capacity; GWG, gestational weight gain*  *Bold represents statistically significant (p ≤ 0.05) findings*  *^a^Models were adjusted for study site, age, race/ethnicity, household income, marital status, education, parity, and pre-pregnancy cigarette smoke exposure. Models for gestational weight gain were additionally adjusted for pre-pregnancy BMI, diabetes, and hypertension.*  *^b^Reference group is BMI < 25*  *^c^For a 1-IQR increase. For subscapular and triceps skinfolds, the IQR is 13.0 millimeters. For the sum of skinfolds, the IQR is 22.5 milimeters.*  *^d^Reference group is adequate gestational weight gain* | | | | | | | | | | |
