# Supplementary material for: Maternal body composition and gestational weight gain in relation to asthma control during pregnancy
Source: PLoS One. 2022 Apr 20;17(4):e0267122. doi: 10.1371/journal.pone.0267122 (PMC9020691; doi:10.1371/journal.pone.0267122)
Supplement: S12 Table — (DOCX) [file pone.0267122.s012.docx]

| S12 Table. **Sensitivity analyses for adjusted^a^ association between maternal pre-pregnancy BMI and gestational weight gain with incidence of asthma-like symptom among women without asthma in the Breathe-Wellbeing, Environment, Lifestyle, and Lung Function Study, 2015-2019, USA.** | | | | | | | | |
| --- | --- | --- | --- | --- | --- | --- | --- | --- |
|  | Activity limitation | | Night symptoms | | Rescue inhaler use | | Respiratory symptoms | |
|  | RR | 95% CI | RR | 95% CI | RR | 95% CI | RR | 95% CI |
| First trimester |  |  |  |  |  |  |  |  |
| BMI 25-30^b^ | 1.17 | 0.35, 3.87 | 1.17 | 0.35, 3.87 | - | - | 1.65 | 0.63, 4.34 |
| BMI ≥ 30^b^ | 1.90 | 0.64, 5.58 | 1.90 | 0.64, 5.58 | - | - | **3.03** | **1.20, 7.62** |
| Subscapular skinfold^c^ | 0.87 | 0.36, 2.06 | 0.76 | 0.38, 1.52 | - | - | 1.30 | 0.70, 2.38 |
| Triceps skinfold^c^ | 0.87 | 0.47, 1.62 | 1.51 | 0.92, 2.48 | - | - | **1.55** | **1.01, 2.36** |
| Sum of skinfolds^c^ | 0.84 | 0.40, 1.76 | 1.27 | 0.66, 2.42 | - | - | 1.70 | 0.98, 2.96 |
| First trimester GWG: inadequate^d^ | 1.17 | 0.35, 3.87 | 1.17 | 0.35, 3.87 | - | - | 1.17 | 0.35, 3.87 |
| First trimester GWG: excessive^d^ | 1.90 | 0.64, 5.58 | 1.90 | 0.64, 5.58 | - | - | 1.90 | 0.64, 5.58 |
| Second trimester |  |  |  |  | - | - |  |  |
| BMI 25-30^b^ | 1.18 | 0.46, 3.02 | 0.75 | 0.27, 2.15 | - | - | 1.01 | 0.28, 3.61 |
| BMI ≥ 30^b^ | 0.53 | 0.14, 2.05 | 1.51 | 0.56, 4.09 | - | - | 2.52 | 0.93, 6.87 |
| Subscapular skinfold^c^ | 0.59 | 0.25, 1.42 | 0.59 | 0.30, 1.18 | - | - | 1.48 | 0.81, 2.71 |
| Triceps skinfold^c^ | 0.68 | 0.35, 1.32 | 1.12 | 0.69, 1.81 | - | - | **1.65** | **1.07, 2.53** |
| Sum of skinfolds^c^ | 0.63 | 0.31, 1.27 | 0.88 | 0.49, 1.57 | - | - | **1.80** | **1.06, 3.05** |
| First trimester GWG: inadequate^d^ | 1.21 | 0.11, 13.11 | 1.55 | 0.40, 5.96 | - | - | 0.56 | 0.16, 1.99 |
| First trimester GWG: excessive^d^ | 0.34 | 0.07, 1.74 | 0.57 | 0.15, 2.19 | - | - | 0.60 | 0.20, 1.81 |
| Second trimester GWG: inadequate^d^ | 8.39 | 0.31, 228.40 | 9.62 | 0.43, 214.42 | - | - | 2.28 | 0.38, 13.67 |
| Second trimester GWG: excessive^d^ | 18.73 | 0.83, 421.64 | 10.87 | 0.55, 214.95 | - | - | 2.31 | 0.39, 13.87 |
| Third trimester |  |  |  |  | - | - |  |  |
| BMI 25-30^b^ | 1.23 | 0.54, 2.79 | 1.01 | 0.36, 2.87 | - | - | 1.07 | 0.28, 4.03 |
| BMI ≥ 30^b^ | 0.46 | 0.13, 1.59 | 2.12 | 0.77, 5.80 | - | - | 2.66 | 0.97, 7.32 |
| Subscapular skinfold^c^ | 0.44 | 0.19, 1.01 | 0.71 | 0.37, 1.33 | - | - | 1.48 | 0.82, 2.67 |
| Triceps skinfold^c^ | 0.65 | 0.35, 1.20 | 1.16 | 0.73, 1.83 | - | - | **1.74** | **1.14, 2.66** |
| Sum of skinfolds^c^ | 0.54 | 0.28, 1.05 | 0.96 | 0.55, 1.69 | - | - | **1.90** | **1.13, 3.18** |
| First trimester GWG: inadequate^d^ | 2.85 | 0.48, 17.00 | 1.64 | 0.42, 6.33 | - | - | 0.54 | 0.17, 1.79 |
| First trimester GWG: excessive^d^ | 0.45 | 0.10, 2.05 | 0.75 | 0.19, 2.93 | - | - | 0.58 | 0.18, 1.88 |
| Second trimester GWG: inadequate^d^ | 1.63 | 0.27, 9.81 | 32.63 | Inestimable | - | - | 1.31 | 0.20, 8.57 |
| Second trimester GWG: excessive^d^ | **5.53** | **1.05, 29.14** | 1.70 | 0.21, 13.96 | - | - | 1.95 | 0.36, 10.63 |
| Third trimester GWG: inadequate^d^ | **0.32** | **0.11, 0.93** | 1.01 | 0.24, 4.33 | - | - | 1.51 | 0.50, 4.53 |
| Third trimester GWG: excessive^d^ | 0.65 | 0.15, 2.74 | 27.57 | Inestimable | - | - | 1.04 | 0.17, 6.48 |
| *Abbreviations: BMI, Body mass index; CI, confidence interval; GWG, gestational weight gain; RR, relative rate ratio*  *Bold represents statistically significant (p ≤ 0.05) findings*  *^a^Models were adjusted for study site, age, race/ethnicity, household income, marital status, education, parity, and pre-pregnancy cigarette smoke exposure. Models for gestational weight gain were additionally adjusted for pre-pregnancy BMI, diabetes, and hypertension.*  *^b^Reference group is BMI < 25*  *^c^For a 1-IQR increase. For subscapular and triceps skinfolds, the IQR is 13.0 millimeters. For the sum of skinfolds, the IQR is 22.5 milimeters.*  *^d^Reference group is adequate gestational weight gain* | | | | | | | | |
