# Supplementary material for: Maternal body composition and gestational weight gain in relation to asthma control during pregnancy
Source: PLoS One. 2022 Apr 20;17(4):e0267122. doi: 10.1371/journal.pone.0267122 (PMC9020691; doi:10.1371/journal.pone.0267122)
Supplement: S1 File — (PDF) [file pone.0267122.s013.pdf]

B-WELL-Mom
Baseline Questionnaire

Study ID: ☐☐☐-☐☐☐-☐☐☐
Site ID Group

Your Characteristics

First we would like to ask you a few basic things about yourself:

1. What is your date of birth? mm/dd/yyyy

2. What is your current address?

   Street:____________________
   City:____________
   State:____ Zipcode:__________

   State options: 1=Alabama – AL
   2=Illinois – IL
   3=Indiana – IN
   4=Michigan – MI
   5=Wisconsin - WI

3. Which one or more of the following would you say is your race/ethnicity? (Select all that apply):
   a. White
   b. Black or African American
   c. Asian
   d. Native Hawaiian or Other Pacific Islander
   e. American Indian or Alaska Native
   f. Hispanic (IF HISPANIC IS SELECTED, GO TO Q4. IF NOT SELECTED SKIP TO Q5)
   g. Other, please specify:___________

4. Please specify your Hispanic origin or ancestry (select all that apply):
   a. Mexican, Mexican American, Chicano
   b. Central or South American
   c. Cuban/Cuban American
   d. Dominican
   e. Puerto Rican
   f. Other, please specify: ______________________

5. Were you born in the US?
   a. Yes (SKIP TO Q8)
   b. No

6. In what country were you born?
   a. Mexico
   b. Guatemala

    c. Honduras
    d. El Salvador
    e. Other, please specify:_____________________

7. How long have you lived in the US? _ Less than 1 year -> ____# months
   _ 1 year or more -> ____# years

8. What is your current marital status?
    a. Married and/or living with partner
    b. Divorced
    c. Widowed
    d. Separated
    e. Single

9. What is the highest grade or year of school **you** completed?
    a. No formal schooling
    b. Less than high school
    c. Some high school
    d. High school graduate or GED
    e. Some college but no degree
    f. Associate’s Degree (Occupational, Technical or Vocational Program)
    g. Bachelor’s Degree (e.g. BA, BS)
    h. Master’s Degree (e.g. MA, MS, MSW, MEng, MBA)
    i. Advanced Degree (e.g. MD, PhD, EdD, DVM)

10. What is the highest grade or year of school **your mother** completed?
    a. No formal schooling
    b. Less than high school
    c. Some high school
    d. High school graduate or GED
    e. Some college but no degree
    f. Associate’s Degree (Occupational, Technical or Vocational Program)
    g. Bachelor’s Degree (e.g. BA, BS)
    h. Master’s Degree (e.g. MA, MS, MSW, MEng, MBA)
    i. Advanced Degree (e.g. MD, PhD, EdD, DVM)
    j. Don’t know

11. What is the highest grade or year of school **your father** completed?
    a. No formal schooling
    b. Less than high school
    c. Some high school
    d. High school graduate or GED
    e. Some college but no degree
    f. Associate’s Degree (Occupational, Technical or Vocational Program)
    g. Bachelor’s Degree (e.g. BA, BS)
    h. Master’s Degree (e.g. MA, MS, MSW, MEng, MBA)
    i. Advanced Degree (e.g. MD, PhD, EdD, DVM)
    j. Don’t know

Now we will collect some information about your household. These questions help us understand ways that families differ that might be related to their health. Your answers will be combined with others in any reports and your personal information is confidential.

12. How many people (including yourself), both children and adults currently live in your household. Include any persons who usually stay here but are temporarily away on business, vacation, in the hospital, on full-time active military duty, or students living temporarily away from home. Do not include anyone who is living in a nursing home or other institution.

    How many adults 18 years of age or older, including yourself live in your household? ___

    How many children less than 18 years of age currently live in your household? ___

13. During the past year, did anyone in your household receive any of the following benefits or was anyone in your household enrolled in any of the following assistance programs?

a.	Aid to Families with Dependent Children:	Yes	No	Don't know
b.	Food stamps:	Yes	No	Don't know
c.	WIC:	Yes	No	Don't know
d.	Free school lunch programs:	Yes	No	Don't know
e.	Social Security benefits:	Yes	No	Don't know
f.	Supplemental Security Income (SSI) Disability benefits:	Yes	No	Don't know
g.	Other:	Yes	No	Don't know
	Please specify:________________________________			

14. What was the total annual income of your entire household in the past year (include income earned by everyone over 18 years of age)? If you are not sure, give us your best guess.
    a. Less than $5,000 (less than $100 per week)
    b. $5,000 - $10,000 ($100 - $200 per week)
    c. $10,000 - $15,000 ($200 - $300 per week)
    d. $15,000 - $20,000 ($300 - $400 per week)
    e. $20,000 - $30,000 ($400 - $575 per week)
    f. $30,000 - $40,000 ($575 - $750 per week)
    g. $40,000 - $50,000 ($750 - $950 per week)
    h. $50,000 -$60,000 ($950 - $1,150 per week)
    i. $60,000 -$70,000 ($1,150 - $1,350 per week)
    j. $70,000 - $80,000 ($1,350 - $1,550 per week)
    k. $80,000 - $100,000 ($1,550 - $1,900 per week)
    l. $100,000-$120,000 ($1,900 - $2,250 per week)
    m. $120,000-$140,000 ($2,250 - $2,650 per week)
    n. More than $140,000 (more than $2,650 per week)

Places You Have Lived

In these next few questions, we would like to ask you some things about the place where you are currently living:

1. Do you own your current residence?
    a. Yes

b. No

2. Do you know the month and year when you started living in your current address?
    a. Yes
        i. MM/YYYY:_________
    b. Don't know month/year, but can estimate
        i. Number of years
            a. Less than 1 year
            b. 1-20
            c. More than 20
    c. Don't know and can't estimate

(NOTE: IF RESPONDENT HAS LIVED IN THEIR CURRENT ADDRESS 1 YEAR OR MORE OR 'DON'T KNOW AND CAN'T ESTIMATE', THEY WILL NOT BE ASKED QUESTIONS 16-22)

3. What type of residence are you currently living in?
    a. Single house
    b. House attached to one or more other houses (duplex/triplex/4-plex)
    c. Building with 2-4 apartments
    d. Building with 5-19 apartments
    e. Building with 20 or more apartments
    f. Condominium
    g. Mobile home, RV, van
    h. Dormitory or residence hall
    i. Don't know
    j. Other (Please specify ___________)

4. About how old was the building when you moved in (your best estimate)?
    a. More than 50 years old
    b. 25-50 years old
    c. 10-25 years old
    d. Less than 10 years old
    e. Don't know

5. Does the tap water in your current home come from a private well? This is not common.
    a. Yes
    b. No
    c. Don't Know

6. Do you use a gas range or stovetop for cooking in your current home?
    a. Yes
    b. No (SKIP TO Q10)

7. Do you have a hood or vent fan over the stove?
    a. Yes
    b. No (SKIP to Q10)

8. Does the hood or vent fan exhaust air to the outside?
    a. Yes
    b. No
    c. Don't know

9. How often do you use the hood or vent fan over the stove when cooking?
    a. Always
    b. Usually
    c. Sometimes
    d. Rarely
    e. Never

10. Is your current home within ¼ mile of agricultural fields or golf course?
    a. Yes
    b. No
    c. Don't know

11. Is your current home within 3 blocks of a gas station?
    a. Yes
    b. No
    c. Don't know

12. Do you use an ozone air ionizer or purifier in your current home?
    a. Yes
    b. No

13. What type of air conditioning do you use in your current house? (check all that apply)
    a. None
    b. Central air conditioning
    c. Room air conditioning (a window unit, for example)
    d. Other. Specify:_______

14. What is the fuel source used for the heating system in your house? (check all that apply)
    a. None
    b. Natural gas
    c. Oil
    d. Propane
    e. Wood or pellet stove
    f. Fireplace
    g. Coal stove
    h. Electric
    i. Other. Specify:_____

15. *Below is a chart with each season listed. Thinking about the past year, you should fill in the number of hours in a typical day that your home has "no ventilation", "low", "medium" or "high" ventilation for each season using the definitions below to help you. The totals should add up to 24 hours. If you are not sure, give us your best guess.*

    No ventilation: All windows and doors closed.

    Low: One or two windows or doors open just a crack (up to 1 inch).

    Medium: Several windows or doors open at least a crack, or one or two windows open part-way (at least several inches).

High: Some windows or doors fully open, or several windows or doors open part-way, or almost all windows or doors open at least a crack.

(**NOTE**: The number of hours for no ventilation, low, medium, and high **SHOULD TOTAL 24** for each season)

	No ventilation		Low		Medium		High		Total hours per day
a. Summer	__(hours)	+	_(hours)	+	__(hours)	+	__(hours)	=	24(hours)
b. Fall	__(hours)	+	__(hours)	+	__(hours)	+	__(hours)	=	24(hours)
c. Winter	__ (hours)	+	__hours)	+	__(hours)	+	__(hours)	=	24(hours)
d. Spring	__(hours)	+	__(hours)	+	__(hours)	+	__(hours)	=	24(hours)

Now we would like to ask you about the place you lived before you were in your current home.

16. Other than your current address, have you lived at any other address **in the past year**?
    a. Yes
    b. No (SKIP TO Q23)

17. Do you know the month and year when you started living in your previous address?
    a. Yes
        i. Month______ / Year_______
    b. Don't know month/year but can estimate
        i. Number of years _____
            a. Less than 1 year
            b. 1-20
            c. More than 20
    c. Don't know and can't estimate

18. What type of residence was your previous address?
    a. Single house
    b. House attached to one or more other houses (duplex/triplex/4-plex)
    c. Building with 2-4 apartments
    d. Building with 5-19 apartments
    e. Building with 20 or more apartments
    f. Condominium
    g. Mobile home, RV, van
    h. Dormitory or residence hall
    i. Don't know
    j. Other (Please specify ___________)

19. About how old was the building at your previous address when you moved in (your best estimate)?
    a. More than 50 years old
    b. 25-50 years old
    c. 10-25 years old

d. Less than 10 years old
e. Don’t Know

20. Did the tap water at your previous address come from a private well?
    a. Yes
    b. No
    c. Don’t Know

21. Was your previous home within ¼ mile of agricultural fields or golf course?
    a. Yes
    b. No
    c. Don’t Know

22. Was your previous home within 3 blocks of a gas station?
    a. Yes
    b. No
    c. Don’t Know

(NOTE: WE MAY NEED TO REPEAT THIS SET OF QUESTIONS IF WOMEN HAVE MORE THAN 2 HOMES IN THE PAST YEAR.)

*Now we will ask you about some of the characteristics of your **current** home:*

23. Do any pets live in your household?
    a. Yes
    b. No (SKIP TO Q24)

    23a. Please indicate how many of each kind of pet live in your household

i.	Cat:	□No	□Yes →	Number:______
ii.	Dog:	□No	□Yes →	Number:______
iii.	Rabbit:	□No	□Yes →	Number:______
iv.	Other:	□No	□Yes →	Specify:_______ Number:_____
v.	Other:	□No	□Yes →	Specify:_______ Number:_____

24. Has there ever been mold in your current house?
    a. Yes
    b. No (SKIP TO Q25)
    c. Don’t Know (SKIP TO Q25)

    24a. Was the mold in the shower area?
        a. Yes
        b. No
        c. Don’t know

    24b. Was the mold in other parts of the house (walls, ceilings, etc.)?
        a. Yes
        b. No
        c. Don’t know

25. Has your current house ever had a sustained water problem (leaks, flooding, etc.)?
    a. Yes
    b. No
    c. Don’t know

26. Has your current house ever had a problem with roaches or other pests?
    a. No
    b. Yes, occasionally
    c. Yes, most of the time
    d. Don’t know

Jobs You Have Had

This next set of questions will ask you about your current job or work situation:

1. Are you currently a student?
    a. No
    b. Yes, full-time
    c. Yes, part-time

2. Did you have a paid job in the past year?
    a. Yes
    b. No (SKIP to Q4a)

3. Are you currently
    a. Employed full-time (SKIP TO Q5)
    b. Employed part-time (SKIP TO Q5)
    c. On leave from employment such as disability leave (SKIP TO Q5)
    d. Unemployed

4. How many months have you been unemployed? (<1,1-11) months (SKIP to Q5)

    4a. How many years have you been unemployed? _____ years (SKIP to Q17)

5. What is/was your occupation? (DROP DOWN LIST)
    a. Management
    b. Business or financial operations
    c. Computer and Mathematical
    d. Architecture and engineering
    e. Life, physical, and social science
    f. Community and social services
    g. Legal
    h. Education, training, library
    i. Art, design, entertainment
    j. Healthcare practitioner
    k. Healthcare support
    l. Protective service
    m. Food preparation and serving

n. Building and grounds cleaning and maintenance
o. Personal care and service
p. Sales and related
q. Office and administrative support
r. Farming, fishing, forestry
s. Construction
t. Installation, maintenance and repair
u. Production
v. Transportation and material moving
w. Military
x. Student
y. Other (please specify):_________________________________

6. How long have you worked (or did you work) at that type of job?
   _Less than 1 year -> How many months? (<1-11)
   _1 year or more -> How many years?____

7. Which of the following best describes or described your usual work schedule (check one)?
   a. Day shift
   b. Afternoon shift
   c. Night shift
   d. Split shift
   e. Irregular shift/on-call
   f. Rotating shifts

Now we would like to collect some information about your workplace environment where you spend the majority of your working day:

8. Is/was your work environment:
   a. Mostly carpeted
   b. Mostly tiled
   c. Mostly hardwood
   d. Mostly outside
   e. None of the above

9. Is/was it air-conditioned?
   a. Yes
   b. No

10. Is/was smoking permitted?
    a. Yes
    b. No

11. Are/were you exposed to chemicals or strong odors?
    a. Yes → Please tell us more about it: ________________________________
    b. No

12. Is/was your work environment moldy or musty?
    a. Yes
    b. No

13. Have you worked in a building (other than your home) with sustained water problems (leaks, flooding, etc.)?
    a. Yes
    b. No
    c. Don’t know

(IF NO ASTHMA, SKIP TO Q15. QUESTION 14 FOR WOMEN WITH ASTHMA ONLY)

14. Are/were your asthma symptoms worse at work?
    a. Yes
    b. No (SKIP TO Q15)

14a. Which symptoms are worse at work?

i. Wheezing: □No □Yes
ii. Coughing: □No □Yes
iii. Tightness in chest/shortness of breath: □No □Yes
iv. Other : □No □Yes, please specify:______________

15. Have you missed any time from work because of asthma or allergies in the past year?
    a. Yes
    b. No (SKIP TO Q16)

15a. How many days in the past year have you missed work because of asthma or allergies?
________days

Now we would like to ask you about different types of jobs you may have had IN THE PAST YEAR:

16. In the past year, have you worked or been trained in any of the following workplaces or jobs, including part-time or temporary summer employment for at least a month? For each YES, answer for how many months.

ON THE JOB:

a.	Gas station or auto repair shop	NO	YES→How many months (<1-12)
b.	Dry cleaning shop	NO	YES→How many months (<1-12)
c.	Farmer, farmworker or forestry worker	NO	YES→How many months (<1-12)
d.	Laboratory worker	NO	YES→How many months (<1-12)
e.	Housekeeper, maid, janitor or cleaning worker	NO	YES→How many months (<1-12)
f.	Hair stylist or manicurist	NO	YES→How many months (<1-12)
g.	Exterminator or pest control worker	NO	YES→How many months (<1-12)
h.	Taxi or bus driver or other motor vehicle operator	NO	YES→How many months (<1-12)
i.	Parking lot attendant or toll booth operator	NO	YES→How many months (<1-12)
	Veterinarian, animal care worker or poultry or livestock farmer	NO	YES→How many months (<1-12)
j.	Nurse	NO	YES→How many months (<1-12)
k.	Dental assistant	NO	YES→How many months (<1-12)
l.	Flight attendant or pilot	NO	YES→How many months (<1-12)

This next question asks you about things you may have been in contact with either at your job, at home, when doing your favorite hobby or other activity:

17. In the past year, have you had any exposure to or had contact with any of the following at least once a week for at least one month? Check NO or YES for each substance. For each YES, answer for how many months.

a.	Metal or metal compounds, such as lead, mercury, arsenic, boron, chromium, cadmium, and selenium (as filings, dust or fumes)	NO	YES→How many months (<1-12)
b.	Drugs or pharmaceuticals (not for personal use)	NO	YES→How many months (<1-12)
c.	Chemicals used to develop or process photographic film	NO	YES→How many months (<1-12)
d.	Dyes, other than hair dyes	NO	YES→How many months (<1-12)
e.	Grease or oils, such as cutting oil or creosote	NO	YES→How many months (<1-12)
f.	Welding fumes	NO	YES→How many months (<1-12)
g.	Solvents, (chemicals that lubricate, soften or dissolve grease, oil, paints or other materials)	NO	YES→How many months (<1-12)
h.	Chemical fixatives (such as embalming fluids, tissue preservation materials)	NO	YES→How many months (<1-12)
i.	Chemicals to make rubber or plastic	NO	YES→How many months (<1-12)
j.	Pesticides to control insect pests	NO	YES→How many months (<1-12)
k.	Herbicides to control weeds	NO	YES→How many months (<1-12)
l.	Fumigants	NO	YES→How many months (<1-12)
m.	Chemical fertilizers	NO	YES→How many months (<1-12)
n.	Stains, varnish or other wood finishes	NO	YES→How many months (<1-12)
o.	Paints or paint products, or paint thinner or remover	NO	YES→How many months (<1-12)
p.	Natural gas, gasoline or fuel products	NO	YES→How many months (<1-12)
q.	Chemicals to sterilize medical or dental instruments	NO	YES→How many months (<1-12)
r.	Laboratory animals	NO	YES→How many months (<1-12)
s.	Farm animals	NO	YES→How many months (<1-12)

Travel Between Your Home And Work

In this next series of questions we would like to get some information about how you get around town on a typical day, either to go to work, or to school, or to do your usual daily activities:

1. Thinking about your typical daily commute, which of the following BEST describes your usual means of commuting to work/school/other activity? (If you use more than one mode of transportation on your usual daily commute, please check all that apply)
    a. I drive alone
    b. I carpool/drive with others
    c. I ride a motorcycle/scooter
    d. I take a bus
    e. I take a train
    f. I walk

g. I ride a bicycle
h. Other, please specify _______

2. On average, approximately how many minutes does it take you to get to work/school/other activity on a normal day?
    a. <15 min
    b. 15-30 min
    c. 31-45 min
    d. 46-60 min
    e. 61-75 min
    f. 76-90 min
    g. >90 min

3. On average, approximately how far is your commute?
    a. <1 mile
    b. 1-5 miles
    c. 6-10 miles
    d. >10 miles

Body Measurements

Now we would like to ask you some questions about your weight and physical shape:

1. Do you know how much you weighed the month you got pregnant?
    a. Yes
        i. What was your weight: ____ lbs
            1. How certain are you in this response?
                a. Not sure at all
                b. Fairly unsure
                c. Fairly certain
                d. Certain
    b. No

2. Since 18 years of age, do you know what is the <u>most</u> that you have ever weighed not including pregnancy and the 12 months following pregnancy?
    a. Yes
        i. What was the most you weighed: ___ lbs
        ii. How old were you when you weighed that amount? ___years
    b. No

3. Since 18 years of age, do you know what is the least that you have ever weighed?
    a. Yes
        i. What is the least you have weighed ____ lbs
        ii. How old were you when you weighed that amount? ____years
    b. No

4. This is a picture of typical female body figures. Please select the woman that best captures your body figure from age 15 years until now. For each age grouping (up to your current age), give the number of the figure that best describes you at this age.

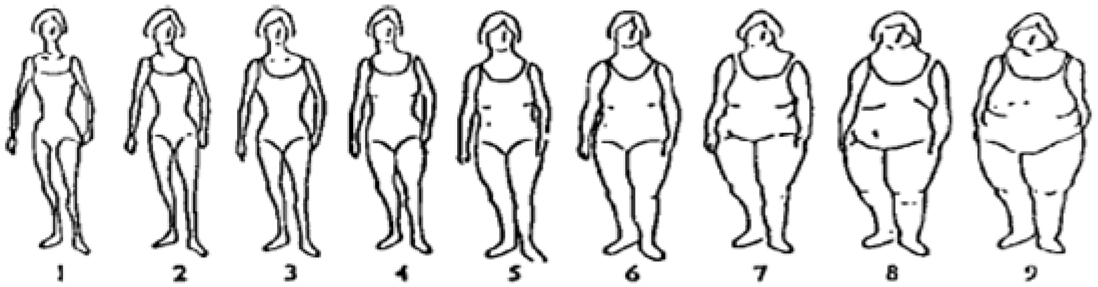

Age (yrs)									
15 to 19	①	②	③	④	⑤	⑥	⑦	⑧	⑨
20 to 24	①	②	③	④	⑤	⑥	⑦	⑧	⑨
25 to 29	①	②	③	④	⑤	⑥	⑦	⑧	⑨
30 to 34	①	②	③	④	⑤	⑥	⑦	⑧	⑨
35 to 39	①	②	③	④	⑤	⑥	⑦	⑧	⑨
40 to 44	①	②	③	④	⑤	⑥	⑦	⑧	⑨
45 to 49	①	②	③	④	⑤	⑥	⑦	⑧	⑨
50 to 55	①	②	③	④	⑤	⑥	⑦	⑧	⑨

Your Health And Lifestyle

Now we would like to ask you some general questions about your health and lifestyle:

1. How is your health, in general?
   a. Excellent
   b. Very Good
   c. Good
   d. Fair
   e. Poor

2. In the month before you got pregnant, how many cigarettes did you smoke on an average day?
   a. None
   b. 1-5
   c. 6-15
   d. 16-20
   e. More than 20

3. How many cigarettes do you smoke on an average day now?
   a. None
   b. 1-5

c. 6-15
d. 16-20
e. More than 20

4. In the month before you got pregnant, was/were there any other household member(s) smoking in your home?
    a. Yes
    b. No

5. Since you got pregnant, is/are there any other household member(s) smoking in your home?
    a. Yes
    b. No

6. In the month before you got pregnant, how many alcoholic drinks (including wine, beer, drinks containing hard liquor, wine coolers, hard lemonade or hard cider) did you consume during an average week?
    a. None
    b. 1-2
    c. 3-4
    d. 5-6
    e. 7 or more

7. In the month before you got pregnant, did you ever drink more than 5 drinks in one occasion?
    a. Yes
    b. No (SKIP TO Q9)

8. How many times in the month before you got pregnant did you drink more than 5 drinks in one occasion?
    a. 1-2
    b. 3-4
    c. 5-6
    d. 7 or more

9. How many alcoholic drinks do you have during an average week now?
    a. None
    b. 1-2
    c. 3-4
    d. 5-6
    e. 7 or more

10. Since you've been pregnant, did you ever drink more than 5 drinks in one occasion?
    a. Yes
    b. No

11. Before you were pregnant, did you regularly take a vitamin or mineral supplement?
    a. Yes
    b. No (SKIP to Q13)

12. How often did you take these vitamins or mineral supplements?

a. Daily
b. Weekly
c. Occasionally

13. Since you learned you were pregnant, do you regularly take a vitamin or mineral supplement?
    a. Yes
    b. No (SKIP to Q15)

14. How often do you take these vitamins or mineral supplements?
    a. Daily
    b. Weekly
    c. Occasionally

15. Before you were pregnant, did you regularly drink caffeinated beverages (coffee, tea, cola, energy drinks? (Do not include decaf coffee)
    a. Yes
    b. No (SKIP to Q17)

16. On a typical day before you were pregnant, how many drinks did you consume:
    a. Caffeinated coffee: number of 8 ounce cups (about the size of a mug)____ (0-10, More than 10)
    b. Caffeinated tea: number of 8 ounce cups (about the size of a mug) ___(0-10, More than 10)
    c. Cans (12 oz) or bottles (16 oz) of soda with caffeine (for example, Coke, Pepsi, Dr. Pepper, Mountain Dew. Count diet and regular together): _____ (0-10, More than 10)
    d. Cans (12 oz) or bottles (16 oz) of energy drinks with caffeine (for example, Red Bull, Amp): _____ (0-10, More than 10)

17. Since you learned you were pregnant, do you drink caffeinated beverages? (Do not include decaf coffee)
    a. Yes
    b. No (SKIP TO Q20)

18. On average, how often do you drink them?
    a. Daily
    b. Weekly
    c. Occasionally

19. On a typical occasion since you learned you were pregnant, when you drank caffeinated beverages, how many drinks did you consume: (if you drank some every day, answer for a typical day)
    a. Caffeinated coffee: number of 8 ounce cups (about the size of a mug) ___(0-10, More than 10)
    b. Caffeinated tea: number of 8 ounce cups (about the size of a mug) ___ (0-10, More than 10)
    c. Cans (12 oz) or bottles (16 oz) of soda with caffeine (for example, Coke, Pepsi, Dr. Pepper, Mountain Dew. Count diet and regular together): ___ (0-10, More than 10)
    d. Cans (12 oz) or bottles (16 oz) of energy drinks with caffeine (for example, Red Bull, Amp): ____ (0-10, More than 10)

20. Has a doctor ever told you that you have (select all that apply):
    a. Depression
    b. Anxiety
    c. Hypertension
    d. Diabetes
    e. Fibromyalgia
    f. Eczema
    g. Gastrointestinal problems (irritable bowel, colitis, Crohn's disease, etc.)
    h. None of the above

21. In a typical year since becoming an adult, do you know about how many colds or upper respiratory infections you get?
    a. Yes
        i. How many: ____Colds per year
    b. No

22. Do you typically get a flu shot each year?
    a. Yes
    b. No

23. Did you get a flu shot in the past year?
    a. Yes
    b. No

24. Were you treated in an emergency department in the last year?
    a. Yes
    b. No (SKIP TO Q25)

    24a. For what reason were you treated in an emergency department in the last year?

    Please specify:_________________________________

25. Were you admitted to a hospital in the last year?
    a. Yes
    b. No (SKIP TO NEXT SECTION)

    25a. For what reason were you admitted to a hospital in the last year?

    Please specify:_________________________________

Reproductive History

This next set of questions is about your reproductive history and current pregnancy:

1. How old were you when you had your first menstrual period?
    i. ____ years
    ii. If not sure, what grade in school were you? ______ grade

2. Not counting this pregnancy, how many times have you been pregnant before (including miscarriages, stillbirths, and abortions)? _____(number)(IF ANSWER IS 0 THEN SKIP TO 12)

3. How old were you at the time of your first pregnancy? ___ years old

4. How many miscarriages have you had? ______number

5. How many times have you had an abortion? _______number

6. How many times have you had a stillbirth? _____number

7. How many live births have you had? _____number (IF ANSWER IS 0 THEN SKIP TO 12)

Now we would like to get some more information about your other children:

8. Were any of your children born three or more weeks early?
   a. Yes
   b. No

9. Did any of your children weigh less than five and a half pounds at birth?
   a. Yes
   b. No

10. Did you breastfeed any of your previous child(ren)?
    a. Yes
    b. No (SKIP to Q12)

11. On average, how long did you breastfeed your other child(ren)?
    a. Less than 12 months
       i. If less than 12 months, how many: ____ months
    b. 1 year or more
       i. If 1 year or more, how many: ____ years ___months
    c. Don't know

12. Do you plan to breastfeed this child?
    a. Yes
    b. No

13. Have you ever tried to become pregnant for more than 12 months without success?
    a. Yes
    b. No

14. Have you ever used oral contraception (the pill), patches, injections, or hormonal IUD for birth control?
    a. Yes
    b. No (SKIP TO Q17)

15. How old were you when you first started using any of these hormonal contraceptives? _____years old

16. How many years total did you use a hormonal contraceptive? _____ years

17. Were you using any type of birth control at the time got pregnant (this pregnancy)?
    a. Yes
    b. No

18. Was your current pregnancy
    a. Planned
    b. Unplanned (SKIP TO Q20)

19. How many menstrual cycles did you try before getting pregnant?
    a. Less than 1 full cycle, got pregnant right away
    b. 1
    c. 2
    d. 3
    e. 4
    f. 5
    g. 6
    h. 7-10
    i. 10-12
    j. More than 12

Now we would like to know a little bit about you when you were born. Some people may have a hard time remembering this information if you were told it a long time ago. Just tell us your best guess, or mark "Don't know" if you really have no idea:

20. Do you know how much you weighed when you were born?
    a. Yes, in lbs
        i. __lbs ___oz
    b. Yes, in grams
        i. _____grams
    c. No

21. Were you born
    a. Early
        i. How many weeks early? ____ weeks
    b. Late
    c. On time
    d. Don't know

22. Were you breastfed as an infant?
    a. Yes→ For how long were you breastfed?
        i. Less than 12 months
            1. How many: ___ months
        ii. 1 year or more

1. How many: ___ years ___ months
iii. Don't know
b. No
c. Don't know

Asthma History (women with asthma only)

Now we would like to ask you some general questions about your asthma:

1. How old were you when you were first told by a doctor or health professional that you had asthma?
   a. ____years _ Age is an estimate

2. Where did you live when you were diagnosed with asthma? 0 US 0 Country outside of US

   If in US, please enter city and select state:

   ______(City) ______(State)

   If outside US, please enter country: ________

3. How long has it been since you last took asthma medication, including a rescue inhaler?
   a. Less than one day ago
   b. 1-6 days ago
   c. 1 week to less than 3 months ago
   d. 3 months to a year ago
   e. Don't know

4. Do you use your inhaler to prevent symptoms when you exercise? (Do you have exercise induced asthma?)
   a. Yes
   b. No
   c. Don't exercise

5. Do you have allergies that make your asthma worse?
   a. Yes
   b. No
   c. Don't know

6. Do any of the following aggravate your asthma?
   a. Respiratory infections:
      i. Yes
      ii. No
      iii. Don't know
   b. Irritants (for example, smoke or chemicals):
      i. Yes
      ii. No
      iii. Don't know
   c. Emotions (crying, anger, etc.):
      i. Yes

    ii. No
    iii. Don’t know

d. Drugs (for example aspirin such as Bayer, or Excedrin,, NSAIDs such as Advil, Aleve, or Motrin, beta-blockers such as Propranolol, Metoprolol, or Atenolol, or ACE-inhibitors such as Lisinopril, Enalapril, Captopril):
    i. Yes
    ii. No
    iii. Don’t know
e. Food additives:
    i. Yes
    ii. No
    iii. Don’t know
f. Weather changes:
    i. Yes
    ii. No
    iii. Don’t know
g. Exercise:
    i. Yes
    ii. No
    iii. Don’t know
h. Cleaning supplies:
    i. Yes
    ii. No
    iii. Don’t know
i. Exposure to animals:

	**Yes**	**No**	**Don’t know**
i. Cat:	◻	◻	◻
ii. Dog:	◻	◻	◻
iii. Rabbit:	◻	◻	◻
iv. Hamster:	◻	◻	◻
v. Guinea pig:	◻	◻	◻
vi. Other rodent:	◻	◻	◻
vii. Other animal:	◻	◻	◻

1. Please specify:___________________

j. A particular season:

	**Yes**	**No**	**Don’t know**
i. Winter:	◻	◻	◻
ii. Spring:	◻	◻	◻
iii. Summer:	◻	◻	◻
iv. Fall:	◻	◻	◻

k. Pre-pregnancy menstrual cycles:

	**Yes**	**No**	**Don’t know**
i. Menses (bleeding)	◻	◻	◻
ii. Premenstrual (week before)	◻	◻	◻
iii. Other times	◻	◻	◻

(Question below is not displayed if woman was not pregnant before.)

7. If you have been pregnant before, did pregnancy usually make your asthma?
    a. Better
    b. Worse
    c. Stayed the same

8. How long has it been since you last had any symptoms of asthma?
    a. Less than one day ago
    b. 1-6 days ago
    c. 1 week to less than 3 months ago
    d. 3 months to a year ago
    e. Don’t know

9. In general, over the last 3 months, how often did you have the following symptoms:

	Symptom	Never	Once a month	1-2 times a week	3-6 times a week	Daily	Twice a day or more
a.	Cough – deep, chest, chronic	☐	☐	☐	☐	☐	☐
b.	Sputum – phlegm or mucus while coughing	☐	☐	☐	☐	☐	☐
c.	Chest tightness – difficulty taking a deep breath or pressure in the chest	☐	☐	☐	☐	☐	☐
d.	Wheezy, whistling, or musical sound in the chest	☐	☐	☐	☐	☐	☐
e.	Shortness of breath	☐	☐	☐	☐	☐	☐
f.	Nighttime symptoms – includes waking from sleep, nighttime use of albuterol, early morning chest tightness	☐	☐	☐	☐	☐	☐

In the next few questions, we would like to know more about your asthma symptoms and recent episodes:

10. Since you have been pregnant, how often do you have any symptoms of asthma?
    a. No symptoms (SKIP TO Q13)
    b. Some days
    c. Most days
    d. Every day
    e. Don’t know

11. Since you’ve been pregnant, do you have symptoms all the time? “All the time” means symptoms that continue throughout the day. It does not mean symptoms for a little while each day.
    a. Yes
    b. No
    c. Don’t know

12. During the past 30 days, on how many nights did symptoms of asthma make it difficult for you to stay asleep?
    a. None
    b. Less than every night
        i. How many: ____ nights (response should be <30 nights)
    c. Every night
    d. Don't know

13. During the past 12 months, have you had an episode of asthma or an asthma attack?
    a. Yes
    b. No (SKIP TO Q17)
    c. Don't know

14. During the past 12 months, how many asthma episodes or attacks have you had? (If you don't know the exact number, just give us your best guess)
    a. ____ episodes

15. How long did your MOST RECENT asthma episode or attack last?
    a. __ __ # of minutes
    b. __ __ # of hours
    c. __ __ # of days
    d. Don't know/Not sure

16. Compared with other episodes or attacks, was this most recent attack shorter, longer, or about the same?
    a. Shorter
    b. Longer
    c. About the same
    d. The most recent attack was actually the first attack
    e. Don't know

Now we would like to ask you about times when you seek treatment for your asthma:

17. During the past 12 months, how many times did you see a doctor or other health professional for a routine checkup for your asthma?
    a. None
    b. 1 time
    c. >1 time
        i. How many: ______
    d. Don't know

18. An urgent care center treats people with illnesses or injuries that must be addressed immediately and cannot wait for a regular medical appointment. During the past 12 months, have you had to visit an emergency room at a hospital or urgent care center because of your asthma?
    a. Yes
    b. No (SKIP TO Q20)
    c. Don't know (SKIP TO Q20)

19. During the past 12 months, how many times did you visit an emergency room or urgent care center because of your asthma?
    a. 1 time
    b. >1 time
        i. How many: _____
    c. Don't know

20. Other than urgent care, during the past 12 months, how many times did you see a doctor or other health professional because of worsening asthma symptoms or for an asthma episode or attack?
    a. None
    b. 1 time
    c. >1 time
        i. How many: ______
    d. Don't know

21. During the past 12 months, have you had to stay overnight in a hospital because of your asthma? Do not include an overnight stay in the emergency room.
    a. Yes
    b. No (SKIP TO Q23)
    c. Don't know

22. During the past 12 months, how many different times did you stay in any hospital overnight or longer because of your asthma? (If you do not know exactly how many, just give us your best guess)
    a. _____ (response should be >0)

23. Over the past 6 months, did you use the following medications/therapies specifically for treatment of asthma:
    a. Inhaled corticosteroids (for example, Beclovent, Pulmicort, Flovent):
        i. No
        ii. Yes→ Which one, please specify________________________________
            1. How often, on average did you take it?
                a. Daily
                b. 2-6 times per week
                c. 1-4 times per month
                d. Less than 1 time per month

    b. Inhaled steroidal combination medications for asthma (for example, Advair, Symbicort):
        i. No
        ii. Yes → Which one, please specify________________________________
            1. How often, on average did you take it?
                a. Daily
                b. 2-6 times per week
                c. 1-4 times per month
                d. Less than 1 time per month

    c. Non-steroidal combination medications for asthma (for example, Combivent):
        i. No
        ii. Yes →Which one, please specify________________________________

1. How often, on average did you take it?
    a. Daily
    b. 2-6 times per week
    c. 1-4 times per month
    d. Less than 1 time per month

d. Oral anti-leukotriene (for example, Singulair, Accolate, Zyflo):
    i. No
    ii. Yes→Which one, please specify________________________________
        1. How often, on average did you take it?
            a. Daily
            b. 2-6 times per week
            c. 1-4 times per month
            d. Less than 1 time per month

e. Inhaled anticholinergic bronchodilators (for example, Atrovent, Spiriva)
    i. No
    ii. Yes →Which one, please specify________________________________
        1. How often, on average did you take it?
            a. Daily
            b. 2-6 times per week
            c. 1-4 times per month
            d. Less than 1 time per month

f. Inhaled short-acting beta agonist bronchodilators (for example, Albuterol,Proventil, Ventolin, Maxair, Xopenex, etc):
    i. No
    ii. Yes→Which one, please specify________________________________
        1. How often, on average did you take it?
            a. Daily
            b. 2-6 times per week
            c. 1-4 times per month
            d. Less than 1 time per month

g. Inhaled long-acting beta-agonist bronchodilators (for example, Serevent, Foradil)
    i. No
    ii. Yes→Which one, please specify________________________________
        1. How often, on average did you take it?
            a. Daily
            b. 2-6 times per week
            c. 1-4 times per month
            d. Less than 1 time per month

h. Cromolyn sodium/nedocromil (for example, Intal, Nasalcrom/Alocril, Tilade):
    i. No
    ii. Yes→Which one, please specify________________________________
        1. How often, on average did you take it?
            a. Daily
            b. 2-6 times per week

            c. 1-4 times per month
            d. Less than 1 time per month

i. Oral beta-agonist (for example, Proventil repetabs)
    i. No
    ii. Yes→Which one, please specify_______________________________
        1. How often, on average did you take it?
            a. Daily
            b. 2-6 times per week
            c. 1-4 times per month
            d. Less than 1 time per month

j. Methylxanthines (for example, theophylline)
    i. No
    ii. Yes→Which one please specify_______________________________
        1. How often, on average did you take it?
            a. Daily
            b. 2-6 times per week
            c. 1-4 times per month
            d. Less than 1 time per month

k. Oral corticosteroid (for example, prednisone pills or liquid):
    i. No
    ii. Yes→Which one, please specify_______________________________
        1. How often, on average did you take it?
            a. Daily
            b. 2-6 times per week
            c. 1-4 times per month
            d. Less than 1 time per month

l. Omalizumab (for example, Xolair)
    i. No
    ii. Yes→Which one, please specify_______________________________
        1. How often, on average did you take it?
            a. 2 times per month
            b. 1 time per month
            c. Less than 1 time per month

m. Steroid injections
    i. No
    ii. Yes→Which one, please specify:_______________________________
        1. If yes, how often, on average did you take it?
            a. Daily
            b. 2-6 times per week
            c. 1-4 times per month
            d. Less than 1 time per month

n. Acupuncture
    i. No

ii. Yes
    1. How often, on average did you receive treatment?
        a. Daily
        b. 2-6 times per week
        c. 1-4 times per month
        d. Less than 1 time per month

o. Allergy shots
    i. No
    ii. Yes→Which one, please specify_______________________________
        1. How often, on average did you take it?
            a. Daily
            b. 2-6 times per week
            c. 1-4 times per month
            d. Less than 1 time per month

p. Chiropractic treatments
    i. No
    ii. Yes
        1. If yes, how often, on average did you receive treatment?
            a. Daily
            b. 2-6 times per week
            c. 1-4 times per month
            d. Less than 1 time per month

q. Herbal or natural treatments, vitamins, etc
    i. No
    ii. Yes→Which one, please specify_______________________________
        1. How often, on average did you take it?
            a. Daily
            b. 2-6 times per week
            c. 1-4 times per month
            d. Less than 1 time per month

r. Other asthma treatment
    i. No
    ii. Yes →Which one, please specify_______________________________
        1. How often, on average did you take it?
            a. Daily
            b. 2-6 times per week
            c. 1-4 times per month
            d. Less than 1 time per month

Allergies

Now we would like to ask you some questions about any allergies you might have:

1. Have you **ever** been told by a doctor or other health care provider that you have any allergies (including medication allergies)?
    a. Yes
    b. No (Skip to Q3)
    c. Don't know

2. What are you allergic to (check all that apply):

		Yes
a.	Foods	◻ → Specify:_______________________
b.	Animals	◻ → Specify:_______________________
c.	Dust	◻
d.	Ragweed or pollen	◻ → Specify:_______________________
e.	Medication	◻ → Specify:_______________________
f.	Other	◻ → Specify:_______________________

3. In the month before you got pregnant did you experience any of the following symptoms? How often did you experience:

**Symptoms**	**Never**	**Less than once a week**	**1 or 2 times a week**	**3 or 4 times a week**	**5 or more times a week**
NASAL					
Runny or Stuffy Nose	◻	◻	◻	◻	◻
Sneezing	◻	◻	◻	◻	◻
Itchy Nose	◻	◻	◻	◻	◻
Nose Bleeds	◻	◻	◻	◻	◻
Loss/Decrease of Sense of Smell	◻	◻	◻	◻	◻
Mouth breathing/Snoring	◻	◻	◻	◻	◻
Sniffing	◻	◻	◻	◻	◻
SINUS					
Headaches	◻	◻	◻	◻	◻
Sore Throats	◻	◻	◻	◻	◻
Post Nasal Drainage	◻	◻	◻	◻	◻
Bad Breath	◻	◻	◻	◻	◻
Hoarseness	◻	◻	◻	◻	◻
Throat Clearing	◻	◻	◻	◻	◻
Itchy Throat	◻	◻	◻	◻	◻
EYE					
Red	◻	◻	◻	◻	◻
Itching	◻	◻	◻	◻	◻
Watery	◻	◻	◻	◻	◻
Dark Circles	◻	◻	◻	◻	◻
Puffiness	◻	◻	◻	◻	◻
EAR					
Full	◻	◻	◻	◻	◻

Painful	◻	◻	◻	◻	◻
Ringing	◻	◻	◻	◻	◻
Hearing Loss	◻	◻	◻	◻	◻
Itching	◻	◻	◻	◻	◻

(NOTE, IF ALL ABOVE ARE “Never”, THEN SKIP TO Q6. IF ANY RESPONSE OTHER THAN “Never”, CONTINUE TO Q4).

4. Are your symptoms:
    a. Year round
    b. Seasonal
    c. Both

5. For each item below, check the box to indicate if your symptoms are worse when exposed to the following. Please select “yes” if your symptoms are ever worsened when exposed.

	Yes	No	Don’t know
a. Changes in season:	◻	◻	◻
b. Changes in weather:	◻	◻	◻
c. Changes in humidity:	◻	◻	◻
d. Dust:	◻	◻	◻
e. Cut grass:	◻	◻	◻
f. Mold or mildew:	◻	◻	◻
g. Pollen:	◻	◻	◻
h. Cat dander:	◻	◻	◻
i. Dog dander:	◻	◻	◻
j. Cigarette smoke:	◻	◻	◻
k. Perfumes:	◻	◻	◻
l. Newsprint:	◻	◻	◻
m. Chemical odors:	◻	◻	◻
n. Exercise:	◻	◻	◻
o. Infections:	◻	◻	◻
p. Emotional stress:	◻	◻	◻

6. Have you ever had allergy skin testing?
    a. Yes
    b. No (SKIP TO Q7)
    c. Don’t know (SKIP TO Q7)

    6a. Were there any positive reactions?
        a. Yes
            a. Please specify:_______________
        b. No
        c. Don’t know

7. Have you ever received allergy injections?
    a. Yes
    b. No (SKIP TO NEXT SECTION)
    c. Don’t know (SKIP TO NEXT SECTION)

    7a. Did your symptoms improve while you received injections?

a. Yes
b. No
c. Don't know

7b. Did you ever experience an adverse reaction to an allergy injection?
a. Yes
    a. Please tell us what reaction you had:_______
b. No
c. Don't know

Physical Activity

1. When you are NOT at work, how much time did you usually spend:

	BEFORE YOU WERE PREGNANT						SINCE GETTING PREGNANT					
	None	Less than ½ hour per day	½ to almost 1 hour per day	1 to almost 2 hours per day	2 to almost 3 hours per day	3 or more hours per day	None	Less than ½ hour per day	½ to almost 1 hour per day	1 to almost 2 hours per day	2 to almost 3 hours per day	3 or more hours per day
a. Preparing meals (cook, set table, wash dishes)	①	②	③	④	⑤	⑥	①	②	③	④	⑤	⑥
b. Dressing, bathing, feeding children while you are sitting	①	②	③	④	⑤	⑥	①	②	③	④	⑤	⑥
c. Dressing, bathing, feeding children while you are standing	①	②	③	④	⑤	⑥	①	②	③	④	⑤	⑥
d. Playing with children while you are sitting or standing	①	②	③	④	⑤	⑥	①	②	③	④	⑤	⑥
e. Playing with children while you are walking or running	①	②	③	④	⑤	⑥	①	②	③	④	⑤	⑥
f. Carrying												

	children	①	②	③	④	⑤	⑥	①	②	③	④	⑤	⑥
g.	Taking care of an older adult	①	②	③	④	⑤	⑥	①	②	③	④	⑤	⑥
h.	Sitting and using a computer or tablet or writing, while <u>not</u> at work	①	②	③	④	⑤	⑥	①	②	③	④	⑤	⑥
i.	Sitting and playing a video game while not at work	①	②	③	④	⑤	⑥	①	②	③	④	⑤	⑥
j.	Playing with pets	①	②	③	④	⑤	⑥	①	②	③	④	⑤	⑥
k.	Light cleaning (make beds, laundry, iron, put things away)	①	②	③	④	⑤	⑥	①	②	③	④	⑤	⑥
l.	Shopping (for food, clothes, or other items)	①	②	③	④	⑤	⑥	①	②	③	④	⑤	⑥
m.	Heavier cleaning (vacuum, mop, sweep, wash windows)	①	②	③	④	⑤	⑥	①	②	③	④	⑤	⑥
n.	Mowing lawn while on a riding mower	①	②	③	④	⑤	⑥	①	②	③	④	⑤	⑥
o.	Mowing lawn while using a walking mower, raking, gardening	①	②	③	④	⑤	⑥	①	②	③	④	⑤	⑥
p.	Walking <u>slowly</u> to go places (such as to the bus, work, visiting) *Not for fun or exercise*	①	②	③	④	⑤	⑥	①	②	③	④	⑤	⑥
q.	Walking <u>quickly</u> to go places (such	①	②	③	④	⑤	⑥	①	②	③	④	⑤	⑥

	as to the bus, work, visiting) *Not for fun or exercise*												
r.	Driving or riding in a car or bus	①	②	③	④	⑤	⑥	①	②	③	④	⑤	⑥
s.	Walking slowly for fun or exercise	①	②	③	④	⑤	⑥	①	②	③	④	⑤	⑥
t.	Walking more quickly for fun or exercise	①	②	③	④	⑤	⑥	①	②	③	④	⑤	⑥
u.	Walking quickly up hills for fun or exercise	①	②	③	④	⑤	⑥	①	②	③	④	⑤	⑥
v.	Jogging	①	②	③	④	⑤	⑥	①	②	③	④	⑤	⑥
w.	Swimming	①	②	③	④	⑤	⑥	①	②	③	④	⑤	⑥
x.	Dancing	①	②	③	④	⑤	⑥	①	②	③	④	⑤	⑥
y.	Doing other things for fun or exercise? Please tell us what they are (or leave this blank if you have none to report). __ __ __	①	②	③	④	⑤	⑥	①	②	③	④	⑤	⑥
z.	Doing other things for fun or exercise? Please tell us up to three activities (or leave this blank if you have none to report). i__________	①	②	③	④	⑤	⑥	①	②	③	④	⑤	⑥
	ii____________	①	②	③	④	⑤	⑥	①	②	③	④	⑤	⑥
	iii____________	①	②	③	④	⑤	⑥	①	②	③	④	⑤	⑥

2. How much time did you usually spend:

	**BEFORE YOU WERE PREGNANT**						**SINCE GETTING PREGNANT**					
	None	Less than ½ hour per day	½ to almost 2 hours per day	2 to almost 4 hours per day	4 to almost 6 hours per day	6 or more hours per day	None	Less than ½ hour per day	½ to almost 2 hours per day	2 to almost 4 hours per day	4 to almost 6 hours per day	6 or more hours per day

a.	Watching TV or a video	①	②	③	④	⑤	⑥	①	②	③	④	⑤	⑥
b.	Sitting and reading, talking or on the phone, **while <u>not</u> at work**	①	②	③	④	⑤	⑥	①	②	③	④	⑤	⑥
c.	Sitting at work or in class	①	②	③	④	⑤	⑥	①	②	③	④	⑤	⑥
d.	Standing or slowly walking **at work** while carrying things (If you were not working, please record "None")	①	②	③	④	⑤	⑥	①	②	③	④	⑤	⑥
e.	Standing or <u>slowly</u> walking **at work** <u>not</u> carrying anything (If you were not working, please record "None")	①	②	③	④	⑤	⑥	①	②	③	④	⑤	⑥
f.	Walking <u>quickly</u> **at work** while <u>carrying</u> things (heavier than a 1 gallon milk jug) (If you were not working, please record "None")	①	②	③	④	⑤	⑥	①	②	③	④	⑤	⑥
g.	Walking <u>quickly</u> **at work** not <u>carrying</u> anything (If you were not working, please record "None")	①	②	③	④	⑤	⑥	①	②	③	④	⑤	⑥

Sleep

Now we would like to get some information about your general sleep patterns every night:

1. How many hours of sleep do you usually get per night:
   a. On weekdays or workdays? (4-10,>10) hours
   b. On weekends? (4-10,>10) hours

2. How many minutes did it usually take for you to fall asleep at bedtime? ____minutes

3. How many minutes of wake time (waking up in the middle of the night) do you have during a typical night's sleep? ____minutes

The next two questions below refer to the times you get in and out of bed to sleep, not including naps.

4. Not including naps, what time did you usually go to bed?
    a. On weekdays or workdays? ___:___ hh:mm AM/PM
    b. On weekends? ___:__ hh:mm AM/PM

5. Not including naps, what time did you usually wake up?
    a. On weekdays or workdays___:___ hh:mm AM/PM
    b. On weekends___:___ hh:mm AM/PM

6. During a usual week, how many times did you nap for 5 minutes or more?
    a. None
    b. 1 or 2 times
    c. 3 or more times

The following questions ask about your sleep habits. Please check one of the following for each of the questions. Pick the answer that best describes how often you experienced the situation.

7. **In the month <u>before</u> you got pregnant**

		**No**	**Yes, Less than once a week**	**Yes, 1 or 2 times a week**	**Yes, 3 or 4 times a week**	**Yes, 5 or more times a week**
a.	Did you have trouble falling asleep?	☐	☐	☐	☐	☐
b.	Did you wake up several times at night?	☐	☐	☐	☐	☐
c.	Did you wake up earlier than you planned to?	☐	☐	☐	☐	☐
d.	Did you have trouble getting back to sleep after you woke up too early?	☐	☐	☐	☐	☐
e.	Did you take sleeping pills to help you sleep?	☐	☐	☐	☐	☐

8. **In the last month**

		**No**	**Yes, Less than once a week**	**Yes, 1 or 2 times a week**	**Yes, 3 or 4 times a week**	**Yes, 5 or more times a week**

a.	Did you have trouble falling asleep?	☐	☐	☐	☐	☐
b.	Did you wake up several times at night?	☐	☐	☐	☐	☐
c.	Did you wake up earlier than you planned to?	☐	☐	☐	☐	☐
d.	Did you have trouble getting back to sleep after you woke up to early?	☐	☐	☐	☐	☐
e.	Did you take sleeping pills to help you sleep?	☐	☐	☐	☐	☐

9. Overall, your typical night's sleep during the last 4 weeks was…
   a. Very sound or restful
   b. Sound or restful
   c. Average quality
   d. Restless
   e. Very restless

10. What position did you usually wake up in?

	**On my left side mostly**	**On my right side mostly**	**Both sides just as much**	**On my back mostly**	**On my front mostly**	**Just as much on my side as on my front or back**	**Sitting up/propped up**	**Don't remember/don't know**
a. Before you got pregnant?	☐	☐	☐	☐	☐	☐	☐	☐
b. During the last week?	☐	☐	☐	☐	☐	☐	☐	☐
c. Last night?	☐	☐	☐	☐	☐	☐	☐	☐

11. How often did you usually wake up during the night?
   a. On average, before you were pregnant? ___times per night
   b. On average, during the last week? ____times per night

c. Last night? ___times per night

12. During the night, how often did you have to get out of bed (for example, to use the toilet)?
    a. On average, before you were pregnant? ___times per night
    b. On average, during the last week? ____times per night
    c. Last night? ___times per night

13. Based on your experience in the last 4 weeks, what is the chance that you would doze off or fall asleep (not just "feel tired") in the following situations? (Check one box for each situation. If you are never or rarely in the situation, please give your best guess for what would happen.)

		**No chance**	**Slight chance**	**Moderate chance**	**High chance**
a.	Sitting and reading?	◻	◻	◻	◻
b.	Watching TV?	◻	◻	◻	◻
c.	Sitting inactive in a public place (such as a theater or meeting)?	◻	◻	◻	◻
d.	Riding as a passenger in a car for an hour without a break?	◻	◻	◻	◻
e.	Lying down to rest in the afternoon when circumstances permit?	◻	◻	◻	◻
f.	Sitting and talking to someone?	◻	◻	◻	◻
g.	Sitting quietly after a lunch without alcohol?	◻	◻	◻	◻
h.	In a car while stopped for a few minutes in traffic?	◻	◻	◻	◻
i.	While driving?	◻	◻	◻	◻

The next set of questions ask about snoring and sleep apnea in the last 4 weeks.

14. In the last 4 weeks, have you snored?
    a. Yes
    b. No (SKIP TO Q18)
    c. Don't know (SKIP TO Q18)

15. In the last 4 weeks, your snoring has been?
    a. Slightly louder than breathing
    b. As loud as talking
    c. Louder than talking
    d. Very loud - could be heard in adjacent rooms

16. In the last 4 weeks, how often have you snored?
    a. Nearly every day
    b. 3-4 times a week
    c. 1-2 times a week

d. 1-2 times a month
e. Nearly never

17. In the last 4 weeks, has your snoring ever bothered other people?
    a. Yes
    b. No
    c. Don't know

18. In the last 4 weeks, has anyone noticed that you quit breathing during your sleep?
    a. Nearly every day
    b. 3-4 times a week
    c. 1-2 times a week
    d. 1-2 times a month
    e. Nearly never
    f. Never

19. In the last 4 weeks, how often did you feel tired or fatigued after your sleep?
    a. Nearly every day
    b. 3-4 times a week
    c. 1-2 times a week
    d. 1-2 times a month
    e. Nearly never
    f. Never

20. During your waking time in the last 4 weeks, did you feel tired, fatigued or not up to par?
    a. Nearly every day
    b. 3-4 times a week
    c. 1-2 times a week
    d. 1-2 times a month
    e. Nearly never
    f. Never

21. In the last 4 weeks, did you ever nod off or fall asleep while driving a vehicle?
    a. Yes
    b. No (SKIP TO NEXT SECTION ON FATIGUE)
    c. Don't drive a vehicle (SKIP TO NEXT SECTION ON FATIGUE)

21a. How often did this occur?
    a. Nearly every day
    b. 3-4 times a week
    c. 1-2 times a week
    d. 1-2 times a month
    e. Nearly never

Now we would like to ask you some questions about fatigue. Fatigue is when you feel weary, tired, or have a lack of energy.

For each of the following questions, indicate the number that most closely indicates how you have been feeling <u>during the past week</u>.

1. To what degree have you experienced fatigue?

☐1 ☐2 ☐3 ☐4 ☐5 ☐6 ☐7 ☐8 ☐9 ☐10

Not at all **A great deal**

If no fatigue (answer choice 1), skip to next section

2. How severe is the fatigue which you have been experiencing?

☐1 ☐2 ☐3 ☐4 ☐5 ☐6 ☐7 ☐8 ☐9 ☐10

Mild **Severe**

3. To what degree has fatigue caused you distress?

☐1 ☐2 ☐3 ☐4 ☐5 ☐6 ☐7 ☐8 ☐9 ☐10

No distress **A great deal of distress**

In the past week, rate on a scale from 1-10 (1=Not at all, 10=A great deal) the degree fatigue has interfered with your ability to:

(NOTE: Check the box to the right of each activity if you don't do the activity)

Fatigue interfered with your ability to:

4. **Do household chores:** Don’t do activity ☐

☐1 ☐2 ☐3 ☐4 ☐5 ☐6 ☐7 ☐8 ☐9 ☐10

Not at all **A great deal**

5. **Cook:** Don’t do activity ☐

☐1 ☐2 ☐3 ☐4 ☐5 ☐6 ☐7 ☐8 ☐9 ☐10

Not at all **A great deal**

6. **Bathe or wash:** Don’t’ do activity ☐

☐1 ☐2 ☐3 ☐4 ☐5 ☐6 ☐7 ☐8 ☐9 ☐10

Not at all **A great deal**

7. **Dress:** Don’t do activity ☐

☐1 ☐2 ☐3 ☐4 ☐5 ☐6 ☐7 ☐8 ☐9 ☐10

Not at all **A great deal**

8. **Work:** Don't do activity☐

☐1 ☐2 ☐3 ☐4 ☐5 ☐6 ☐7 ☐8 ☐9 ☐10

Not at all **A great deal**

9. **Visit or socialize with friends or family**: Don't do activity ☐

☐1 ☐2 ☐3 ☐4 ☐5 ☐6 ☐7 ☐8 ☐9 ☐10

Not at all **A great deal**

10. **Engage in sexual activity:** Don't do activity ☐

☐1 ☐2 ☐3 ☐4 ☐5 ☐6 ☐7 ☐8 ☐9 ☐10

Not at all **A great deal**

11. **Engage in leisure and recreational activities**: Don't do activity ☐

☐1 ☐2 ☐3 ☐4 ☐5 ☐6 ☐7 ☐8 ☐9 ☐10

Not at all **A great deal**

12. **Shop and do errands**: Don't do activity ☐

☐1 ☐2 ☐3 ☐4 ☐5 ☐6 ☐7 ☐8 ☐9 ☐10

Not at all **A great deal**

13. **Walk:** Don't do activity ☐

☐1 ☐2 ☐3 ☐4 ☐5 ☐6 ☐7 ☐8 ☐9 ☐10

Not at all **A great deal**

14. **Exercise, other than walking:** Don't do activity ☐

☐1 ☐2 ☐3 ☐4 ☐5 ☐6 ☐7 ☐8 ☐9 ☐10

Not at all **A great deal**

15. Over the past week, how often have you been fatigued?

a. Every day
b. Most, but not all days
c. Occasionally, but not most days
d. Hardly any days

16. To what degree has your fatigue changed during the past week?
a. Increased
b. Fatigue has gone up and down
c. Stayed the same
d. Decreased

Health

Now we would like to ask you some questions about your emotions and how you've been feeling lately .There are no "right" or "wrong" answers to any of these questions.

For the following 20 items, please select the choice that best describes how you have felt ***over the past week*****:**

		**Rarely (< 1 day) or never**	**Some or a little of the time (1-2 days)**	**Occasionally or a moderate amount of the time (3-4 days)**	**Most or all of the time (5-7 days)**
1	I was bothered by things that don't usually bother me.	①	②	③	④
2	I did not feel like eating; my appetite was poor.	①	②	③	④
3	I felt that I could not shake off the blues even with the help from my family and friends.	①	②	③	④
4	I felt that I was not as good as other people.	①	②	③	④
5	I had trouble keeping my mind on what I was doing.	①	②	③	④
6	I felt depressed.	①	②	③	④
7	I felt that everything I did was an effort.	①	②	③	④
8	I felt hopeless about the future.	①	②	③	④
9	I thought my life had been a failure.	①	②	③	④

10	I felt fearful.	①	②	③	④
11	My sleep was restless.	①	②	③	④
12	I was unhappy.	①	②	③	④
13	I talked less than usual.	①	②	③	④
14	I felt lonely.	①	②	③	④
15	People were unfriendly.	①	②	③	④
16	I did not enjoy life.	①	②	③	④
17	I had crying spells.	①	②	③	④
18	I felt sad.	①	②	③	④
19	I felt that people disliked me.	①	②	③	④
20	I could not get “going”.	①	②	③	④

With this next set of questions we want to know about your level of anxiety <u>over the last 2 weeks</u>:

1. Over the last 2 weeks, how many days have you been nervous, anxious, or on edge?
    a. 0
    b. 1-2
    c. 3-4
    d. >4
    e. Don't know

2. Over the last 2 weeks, how many days have you not been able to stop or control worrying?
    a. 0
    b. 1-2
    c. 3-4
    d. >4
    e. Don't know

3. Over the last 2 weeks, how many days have you worried too much about different things?
    a. 0
    b. 1-2
    c. 3-4
    d. >4
    e. Don't know

4. Over the last 2 weeks, how many days have you had trouble relaxing?
    a. 0
    b. 1-2
    c. 3-4
    d. >4
    e. Don't know

5. Over the last 2 weeks, how many days have you been so restless that it was hard to sit still?
    a. 0

b. 1-2
c. 3-4
d. >4
e. Don't know

6. Over the last 2 weeks, how many days have you been easily annoyed or irritable?
a. 0
b. 1-2
c. 3-4
d. >4
e. Don't know

7. Over the last 2 weeks, how many days have you felt afraid as if something awful might happen?
a. 0
b. 1-2
c. 3-4
d. >4
e. Don't know

Now we would like to know about stress in your life in the last month.

For each question, please check the answer that is most true for you. There are no "right" or "wrong" answers.

		**Never (1)**	**Almost Never (2)**	**Some-times (3)**	**Fairly Often (4)**	**Often (5)**
1	In the last month, how often have you been upset because of something that happened unexpectedly?	①	②	③	④	⑤
2	In the last month, how often have you felt that you were unable to control the important things in your life?	①	②	③	④	⑤
3	In the last month, how often have you felt nervous and “stressed’?	①	②	③	④	⑤
4	In the last month, how often have you dealt successfully with irritating life hassles?	①	②	③	④	⑤
5	In the last month, how often have you felt that things were going your way?	①	②	③	④	⑤
6	In the last month, how often have you found you could not cope with all the things that you had to do?	①	②	③	④	⑤

7	In the last month, how often have you been able to control irritations in your life?	①	②	③	④	⑤
8	In the last month, how often have you felt that you were on top of things?	①	②	③	④	⑤
9	In the last month, how often have you been angered because of things that happened that were outside of your control?	①	②	③	④	⑤
10	In the last month, how often have you felt difficulties were piling up so high that you could not overcome them?	①	②	③	④	⑤

Father Questions

Now we would like to ask you some of the same questions about your baby's biological father:

Can you answer specific questions about the baby's biological father?

a. Yes
b. No (Skip remainder of section)

1. Do you know how old the biological father of your baby is?
    a. Yes
        i. What is his age? ___ years
    b. No

2. Which one or more of the following would you say is the race/ethnicity of the biological father of your baby? (Select all that apply)
    a. White
    b. Black or African American
    c. Asian
    d. Native Hawaiian or Other Pacific Islander
    e. American Indian or Alaska Native
    f. Hispanic (IF HISPANIC IS SELECTED, GO TO Q3. IF NOT SELECTED SKIP TO Q4)
    g. Other, please specify___________
    h. Don't know

3. Please specify Hispanic origin or ancestry of the biological father of your baby (Select all that apply)
    a. Mexican, Mexican American, Chicano
    b. Central or South American
    c. Cuban/Cuban American
    d. Dominican
    e. Puerto Rican
    f. Other, please specify: ______________________
    g. Don't know

4. What is the highest grade or year of school completed by the biological father of your baby?
    a. No formal schooling
    b. Less than high school
    c. Some high school
    d. High school graduate or GED
    e. Some college but no degree
    f. Associate's Degree (Occupational, Technical or Vocational Program)
    g. Bachelor's Degree (e.g. BA, BS)
    h. Master's Degree (e.g. MA, MS, MSW, MEng, MBA)
    i. Advanced Degree (e.g. MD, PhD, EdD, DVM)
    j. Don't know

5. Do you know approximately how tall the baby's biological father is?
    a. Yes
        a. __ft __in
    b. No

6. How certain are you in this response?
    a. Not sure at all
    b. Fairly unsure
    c. Fairly certain
    d. Certain

7. This is a picture of typical male body figures. Please select the man that best captures your baby's biological father's body now. Give the number of the figure that best describes him at his current age.
    a. 1
    b. 2
    c. 3
    d. 4
    e. 5
    f. 6
    g. 7
    h. 8
    i. 9
    j. Don't know

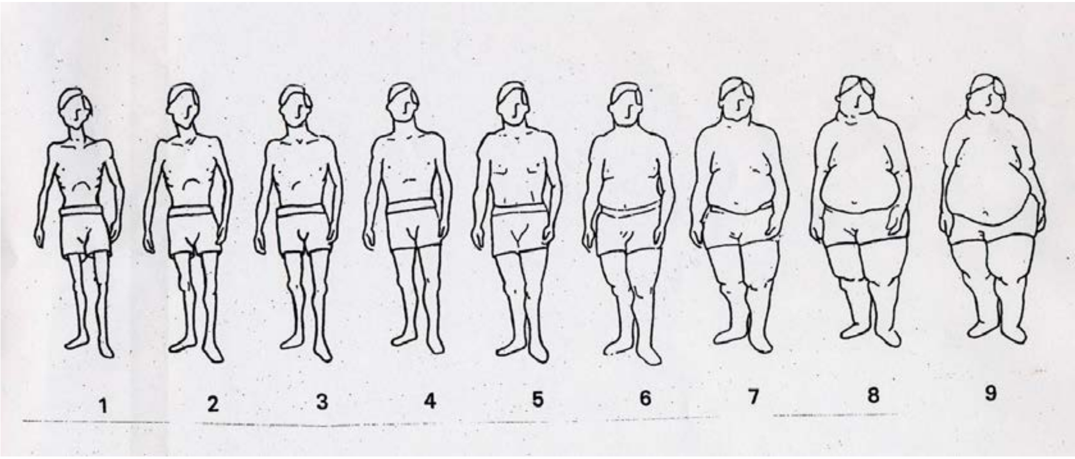

8. Is the biological father of your baby living in the same household with you?
   a. Yes
   b. No

(NOTE: IF MOM HAS OTHER CHILDREN, ASK 9; OTHERWISE SKIP)

9. Is this your first baby with this father? (If he is the father of your other child(ren), answer "No")
   a. Yes
   b. No
   c. Don't know

10. How many cigarettes does your baby's biological father smoke on an average day?
   a. None
   b. 1-5
   c. 6-15
   d. 16-20
   e. More than 20
   f. Don't know

11. Is the baby's biological father currently a student?
   a. No
   b. Yes, full-time
   c. Yes, part-time
   d. Don't know

12. Did your baby's biological father have a job in the past year?
   a. Yes
   b. No (SKIP TO 14a.)
   c. Don't know (SKIP TO Q15)

13. Is he currently unemployed?
   a. Yes
   b. No (SKIP TO 15)

14. How many months has he been unemployed? (<1-11) months (SKIP to Q15)

14a. How many years has he been unemployed? __ years (SKIP to Q17)

15. Do you know what his occupational field is/was?
   a. Yes
   b. No (SKIP TO 16)

15a. What is/was his most recent occupational field? (drop down)
   a. Management
   b. Business or financial operations
   c. Computer and Mathematical
   d. Architecture and engineering
   e. Life, physical, and social science

f. Community and social services
g. Legal
h. Education, training, library
i. Art, design, entertainment
j. Healthcare practitioner
k. Healthcare support
l. Protective service
m. Food preparation and serving
n. Building and grounds cleaning and maintenance
o. Personal care and service
p. Sales and related
q. Office and administrative support
r. Farming, fishing, forestry
s. Construction
t. Installation, maintenance and repair
u. Production
v. Transportation and material moving
w. Military
x. Student
y. Other (please specify):________________________________

16. In the past year, has your baby's biological father worked or been trained in any of the following workplaces or jobs, including part-time or temporary summer employment for at least a month? For each YES, answer for how many months.

ON THE JOB:

c.	Gas station or auto repair shop	NO	YES→How many months? (<1-12)	Don't know
d.	Dry cleaning shop	NO	YES→How many months? (<1-12)	Don't know
e.	Farmer, farmworker or forestry worker	NO	YES→How many months? (<1-12)	Don't know
f.	Laboratory worker	NO	YES→How many months? (<1-12)	Don't know
g.	Housekeeper, janitor or cleaning worker	NO	YES→How many months? (<1-12)	Don't know
h.	Hair stylist or manicurist	NO	YES→How many months? (<1-12)	Don't know
i.	Exterminator or pest control worker	NO	YES→How many months? (<1-12)	Don't know
j.	Taxi or bus driver or other motor vehicle operator	NO	YES→How many months? (<1-12)	Don't know
k.	Parking lot attendant or toll booth operator	NO	YES→How many months? (<1-12)	Don't know
l.	Veterinarian, animal care worker or	NO	YES→How many months? (<1-12)	Don't know
m.	Nurse	NO	YES→How many months? (<1-12)	Don't know
n.	Dental assistant	NO	YES→How many months? (<1-12)	Don't know
o.	Flight attendant or pilot	NO	YES→How many months? (<1-12)	Don't know

This next question asks you about things your baby's biological father may have been in contact with either at his job, at home, when doing his favorite hobby or other activity:

17. In the past year, has your baby's biological father had any exposure to or had contact with any of the following at least once a week for at least one month? Check NO or YES or DON'T KNOW for each substance. For each YES, answer for how many months.

a.	Metal or metal compounds, such as lead, mercury, arsenic, boron, chromium, cadmium, and selenium (as filings, dust or fumes)	NO	YES→How many months (<1-12)	Don't Know
b.	Drugs or pharmaceuticals (not for personal use)	NO	YES→How many months (<1-12)	Don't Know
c.	Chemicals used to develop or process photographic film	NO	YES→How many months (<1-12)	Don't Know
d.	Dyes, other than hair dyes	NO	YES→How many months (<1-12)	Don't Know
e.	Grease or oils, such as cutting oil or creosote	NO	YES→How many months (<1-12)	Don't Know
f.	Welding fumes	NO	YES→How many months (<1-12)	Don't Know
g.	Solvents, (chemicals that lubricate, soften or dissolve grease, oil, paints or other materials)	NO	YES→How many months (<1-12)	Don't Know
h.	Chemical fixatives (such as embalming fluids, tissue preservation materials)	NO	YES→How many months (<1-12)	Don't Know
i.	Chemicals to make rubber or plastic	NO	YES→How many months (<1-12)	Don't Know
j.	Pesticides to control insect pests	NO	YES→How many months (<1-12)	Don't Know
k.	Herbicides to control weeds	NO	YES→How many months (<1-12)	Don't Know
l.	Fumigants	NO	YES→How many months (<1-12)	Don't Know
m.	Chemical fertilizers	NO	YES→How many months (<1-12)	Don't Know
n.	Stains, varnish or other wood finishes	NO	YES→How many months (<1-12)	Don't Know
o.	Paints or paint products, or paint thinner or remover	NO	YES→How many months (<1-12)	Don't Know
p.	Natural gas, gasoline or fuel products	NO	YES→How many months (<1-12)	Don't Know
q.	Chemicals to sterilize medical or dental instruments	NO	YES→How many months (<1-12)	Don't Know
r.	Laboratory animals	NO	YES→How many months (<1-12)	Don't Know
s.	Farm animals	NO	YES→How many months (<1-12)	Don't Know
