# Supplementary material for: Maternal body composition and gestational weight gain in relation to asthma control during pregnancy
Source: PLoS One. 2022 Apr 20;17(4):e0267122. doi: 10.1371/journal.pone.0267122 (PMC9020691; doi:10.1371/journal.pone.0267122)
Supplement: S2 File — (PDF) [file pone.0267122.s014.pdf]

B-WELL-Mom
Clinic Visit 2 and 3 Questionnaire

Study ID: ☐☐☐-☐☐☐-☐☐☐
Site ID Group

Changes To Your Address

First, we would like to ask you if anything has changed about your living situation since your last study visit:

1. Have you moved from this address? _____ (autofill address)?
   a. Yes (SKIP to next section)
   b. No

2. Has there been any change in the number or type of your household pets since your last study visit?
   a. No
   b. Yes → (SKIP TO Q2a.)
   2a. Please indicate how many of each kind of pet you have:

i. Cat:	□No	□Yes →	Number:______	
ii. Dog:	□No	□Yes →	Number:______	
iii. Rabbit:	□No	□Yes →	Number:______	
iv. Other:	□No	□Yes →	Specify:______	Number:____
v. Other:	□No	□Yes →	Specify:______	Number:____

3. Any change in your marital status?
   a. Yes
   b. No (SKIP TO NEXT SECTION ON JOB STATUS)

4. What is your current marital status? Are you:
   a. Married and/or living with partner
   b. Divorced
   c. Widowed
   d. Separated
   e. Single

Details About Your New Home

1. What is your current address?

Street:____________________
City:____________
State:____ Zipcode:__________

State options: 1=Alabama – AL
2=Illinois – IL
3=Indiana – IN
4=Michigan – MI
5=Wisconsin - WI

2. When did you move to your current address?
    a. MM/YYYY: ________

3. What type of residence are you currently living in?
    a. Single house
    b. House attached to one or more other houses (duplex/triplex/4-plex)
    c. Building with 2-4 apartments
    d. Building with 5-19 apartments
    e. Building with 20 or more apartments
    f. Condominium
    g. Mobile home, RV, van.
    h. Dormitory or residence hall
    i. Don't know
    j. Other (Please specify ___________)

4. About how old was the building when you moved in (your best estimate)?
    a. More than 50 years old
    b. 25-50 years old
    c. 10-25 years old
    d. Less than 10 years old
    e. Don't Know

5. Does the tap water in your current home come from a private well? This is not common.
    a. Yes
    b. No
    c. Don't Know

6. Do you use a gas range or stovetop for cooking in your current home?
    a. Yes
    b. No (SKIP TO Q10)

7. Do you have a hood or vent fan over the stove?
    a. Yes
    b. No (SKIP to Q10)

8. Does the hood or vent fan exhaust air to the outside?
    a. Yes
    b. No
    c. Don't know

9. How often do you use the hood or vent fan over the stove when cooking?
    a. Always

b. Usually
c. Sometimes
d. Rarely
e. Never

10. Is your current home within ¼ mile of agricultural fields or golf course?
    a. Yes
    b. No
    c. Don't Know

11. Is your current home within 3 blocks of a gas station?
    a. Yes
    b. No
    c. Don't Know

12. Do you use an ozone air ionizer or purifier in your current home?
    a. Yes
    b. No

13. What type of air conditioning do you use in your current house? (check all that apply)
    a. None
    b. Central air conditioning
    c. Room air conditioning (a window unit, for example)
    d. Other, Specify:_______

14. What is the fuel source used for the heating system in your house? (check all that apply)
    a. None
    b. Natural gas
    c. Oil
    d. Propane
    e. Wood or pellet stove
    f. Fireplace
    g. Coal stove
    h. Electric
    i. Other, Specify:_____

15. *Below is a chart with each season listed. Thinking about the past year, you should fill in the number of hours in a typical day that your home has "no ventilation", "low", "medium" or "high" ventilation for each season using the definitions below to help you. The totals should add up to 24 hours. If you are not sure, give us your best guess.*

    No ventilation: All windows and doors closed.

    Low: One or two windows or doors open just a crack (up to 1 inch).

    Medium: Several windows or doors open at least a crack, or one or two windows open part-way (at least several inches).

    High: Some windows or doors fully open, or several windows or doors open part-way, or almost all windows or doors open at least a crack.

(**NOTE**: The number of hours for no ventilation, low, medium, and high **SHOULD TOTAL 24** for each season)

	No ventilation		Low		Medium		High		Total hours per day
a. Summer	___(hours)	+	___(hours)	+	__(hours)	+	__(hours)	=	24 (hours)
b. Fall	___(hours)	+	___(hours)	+	__(hours)	+	__(hours)	=	24 (hours)
c. Winter	___(hours)	+	___(hours)	+	__(hours)	+	__(hours)	=	24 (hours)
d. Spring	___(hours)	+	___(hours)	+	__(hours)	+	__(hours)	=	24 (hours)

16. Has there been any change in the number or type of your household pets since your last study visit?
    a. No
    b. Yes → (SKIP TO Q16a.)
    16a. Please indicate how many of each kind of pet you have:

i.	Cat:	□No	□Yes →	Number:______	
ii.	Dog:	□No	□Yes →	Number:______	
iii.	Rabbit:	□No	□Yes →	Number:______	
iv.	Other:	□No	□Yes →	Specify:______	Number:____
v.	Other:	□No	□Yes →	Specify:______	Number:____

17. Has there ever been mold in your current house?
    a. Yes
    b. No (SKIP TO Q18)
    c. Don't Know (SKIP TO Q18)

    17a. Was the mold in the shower area?
        a. Yes
        b. No
        c. Don't know

    17b. Was the mold in other parts of the house (walls, ceilings, etc.) ?
        a. Yes
        b. No
        c. Don't know

18. Has your current house ever had a sustained water problem (leaks, flooding, etc.)?
    a. Yes
    b. No
    c. Don't know

19. Has your current house had a problem with roaches or other pests?
    a. No
    b. Yes, occasionally
    c. Yes, most of the time
    d. Don't know

20. Any change in your marital status?
    a. Yes
    b. No (SKIP TO NEXT SECTION)

21. What is your current marital status? Are you:
    a. Married and/or living with partner
    b. Divorced
    c. Widowed
    d. Separated
    e. Single

Changes To Your Job Status

Now we would like to ask you if anything has changed about your work situation since your last study visit:

Your previous employment status was: ___________ (auto-populated from previous visit)

(1=Employed full-time; 2=Employed part-time; 3=On leave from employment, such as disability leave; 4=Unemployed)

1. Any change to your occupation since your last study visit?
    a. Yes
    b. No (see skip patterns below)
       [If previously working, full or part-time, and no change then skip to Q12]
       [If previously unemployed and no change then skip to Q15]
       [If previously on leave and no change then skip to Q15]

2. Are you currently
    a. Employed full-time at a new job (SKIP TO Q4)
    b. Employed part-time at a new job (SKIP TO Q4)
    c. On leave from employment (SKIP TO Q15)
    d. Unemployed
    e. Same job location but working more hours (e.g., from part-time to full-time) (SKIP TO Q15)
    f. Same job location but working fewer hours (e.g., from full-time to part-time) (SKIP TO Q15)

3. How many weeks have you been unemployed? (<1,12,>12) weeks (SKIP TO Q15)

These next few questions are about your new job since your last study visit:

4. What is your new occupation? (DROP DOWN LIST)
    a. Management
    b. Business or financial operations
    c. Computer and Mathematical
    d. Architecture and engineering
    e. Life, physical, and social science
    f. Community and social services
    g. Legal
    h. Education, training, library
    i. Art, design, entertainment

j. Healthcare practitioner
k. Healthcare support
l. Protective service
m. Food preparation and serving
n. Building and grounds cleaning and maintenance
o. Personal care and service
p. Sales and related
q. Office and administrative support
r. Farming, fishing, forestry
s. Construction
t. Installation, maintenance and repair
u. Production
v. Transportation and material moving
w. Military
x. Student
y. Other (please specify):________________________________

5. How long have you worked at that type of job? (<1,1-12,>12) weeks

6. Which of the following best describes your usual work schedule?
    a. Day shift
    b. Afternoon shift
    c. Night shift
    d. Split shift
    e. Irregular shift/on-call
    f. Rotating shifts

Now we would like to collect some information about your new workplace environment where you spend the majority of your working day:

7. Is your work environment:
    a. Mostly carpeted
    b. Mostly tiled
    c. Mostly hardwood
    d. Mostly outside
    e. None of the above

8. Is it air-conditioned?
    a. Yes
    b. No

9. Is smoking permitted?
    a. Yes
    b. No

10. Are you exposed to chemicals or strong odors?
    a. Yes → Please tell us more about it: ________________________________
    b. No

11. Is your work environment moldy or musty?
    a. Yes
    b. No

(IF NO ASTHMA, SKIP TO Q13. Q12 FOR WOMEN WITH ASTHMA ONLY)

12. Are your asthma symptoms worse at work?
    a. Yes
    b. No (SKIP TO Q13)

    12a. Which symptoms are worse at work?
    i. Wheezing: □No □Yes
    ii. Coughing: □No □Yes
    iii. Tightness in chest/shortness of breath: □No □Yes
    iv. Other : □No □Yes, please specify:______________

13. Have you missed any time from work because of asthma or allergies since your last study visit?
    a. Yes
    b. No (SKIP TO Q14)

    13a. How many days since your last study visit have you missed work because of asthma or allergies? ________days

14. Since your last study visit, have you worked or been trained in any of the following workplaces or jobs, including part-time or temporary summer employment for at least a month? For each YES, answer for how many weeks.

ON THE JOB:

a.	Gas station or auto repair shop	NO	YES→How many weeks (<1,1-12,>12)
b.	Dry cleaning shop	NO	YES→How many weeks (<1,1-12,>12)
c.	Farmer, farmworker or forestry worker	NO	YES→How many weeks (<1,1-12,>12)
d.	Laboratory worker	NO	YES→How many weeks (<1,1-12,>12)
e.	Housekeeper, maid, janitor or cleaning worker	NO	YES→How many weeks (<1,1-12,>12)
f.	Hair stylist or manicurist	NO	YES→How many weeks (<1,1-12,>12)
g.	Exterminator or pest control worker	NO	YES→How many weeks (<1,1-12,>12)
h.	Taxi or bus driver or other motor vehicle operator	NO	YES→How many weeks (<1,1-12,>12)
i.	Parking lot attendant or toll booth operator	NO	YES→How many weeks (<1,1-12,>12)
j.	Veterinarian, animal care worker or poultry or livestock farmer	NO	YES→How many weeks (<1,1-12,>12)
k.	Nurse	NO	YES→How many weeks (<1,1-12,>12)
l.	Dental assistant	NO	YES→How many weeks (<1,1-12,>12)
m.	Flight attendant or pilot	NO	YES→How many weeks (<1,1-12,>12)

This next question asks you about things you may have been in contact with either at your job, at home, when doing your favorite hobby or other activity:

15. Since your last study visit, have you had any exposure to or had contact with any of the following at least once a week for at least one month? Check NO or YES for each substance. For each YES, answer for how many weeks.

a. Metal or metal compounds, such as lead, mercury, arsenic, boron, chromium, cadmium, and selenium (as filings, dust or fumes) — NO YES→How many weeks (<1,1-12,>12)
b. Drugs or pharmaceuticals (not for personal use) — NO YES→How many weeks (<1,1-12,>12)
c. Chemicals used to develop or process photographic film — NO YES→How many weeks (<1,1-12,>12)
d. Dyes, other than hair dyes — NO YES→How many weeks (<1,1-12,>12)
e. Grease or oils, such as cutting oil or creosote — NO YES→How many weeks (<1,1-12,>12)
f. Welding fumes — NO YES→How many weeks (<1,1-12,>12)
g. Solvents, (chemicals that lubricate, soften or dissolve grease, oil, paints or other materials) — NO YES→How many weeks (<1,1-12,>12)
h. Chemical fixatives (such as embalming fluids, tissue preservation materials) — NO YES→How many weeks (<1,1-12,>12)
i. Chemicals to make rubber or plastic — NO YES→How many weeks (<1,1-12,>12)
j. Pesticides to control insect pests — NO YES→How many weeks (<1,1-12,>12)
k. Herbicides to control weeds — NO YES→How many weeks (<1,1-12,>12)
l. Fumigants — NO YES→How many weeks (<1,1-12,>12)
m. Chemical fertilizers — NO YES→How many weeks (<1,1-12,>12)
n. Stains, varnish or other wood finishes — NO YES→How many weeks (<1,1-12,>12)
o. Paints or paint products, or paint thinner or remover — NO YES→How many weeks (<1,1-12,>12)
p. Natural gas, gasoline or fuel products — NO YES→How many weeks(<1,1-12,>12)
q. Chemicals to sterilize medical or dental instruments — NO YES→How many weeks(<1,1-12,>12)
r. Laboratory animals — NO YES→How many weeks(<1,1-12,>12)
s. Farm animals — NO YES→How many weeks(<1,1-12,>12)

Changes To Your Travel Patterns

In this next series of questions we would like to update the information you told us about how you get around town every day, either to go to work, or to school, or to do your usual daily activities:

1. Have there been any changes to your typical commuting pattern to work/school/other activity?
   a. Yes
   b. No (SKIP TO NEXT SECTION)

2. Thinking about your typical daily commute, which of the following BEST describes your usual means of commuting to work/school/other activity each day? If you use more than one mode of transportation on your usual daily commute, please check all that apply.
   a. I drive alone
   b. I carpool/drive with others
   c. I ride a motorcycle/scooter
   d. I take a bus
   e. I take a train
   f. I walk
   g. I ride a bicycle
   h. Other, please specify:______

3. On average, approximately how many minutes does it take you to get to work/school/other activity on a normal day?
    a. <15 min
    b. 15-30 min
    c. 31-45 min
    d. 46-60 min
    e. 61-75 min
    f. 76-90 min
    g. >90 min

4. On average, approximately how far is your commute?
    a. <1 mile
    b. 1-5 miles
    c. 6-10 miles
    d. >10 miles

Changes To Your Health And Lifestyle

Now we would like to ask you some general questions about your health and lifestyle since your last study visit:

1. How is your health, in general?
    a. Excellent
    b. Very Good
    c. Good
    d. Fair
    e. Poor

2. How many cigarettes do you smoke on an average day now?
    a. None
    b. 1-5
    c. 6-15
    d. 16-20
    e. More than 20

3. Since your last study visit, does anyone smoke in your home?
    a. Yes
    b. No

4. How many alcoholic drinks do you have during an average week now?
    a. None
    b. 1-2
    c. 3-4
    d. 5-6
    e. 7 or more

5. Since your last study visit, did you ever drink more than 5 drinks in one occasion?

    a. Yes
    b. No (SKIP TO Q7)

6. How many times since your last study visit did you drink more than 5 drinks in one occasion?
    a. 1-2
    b. 3-4
    c. 5-6
    d. 7 or more

7. Since your last study visit, did you ever drink any caffeinated beverages? (Do not include decaf coffee)
    a. Yes
    b. No (SKIP TO NEXT SECTION)

8. On average, how often did you drink them?
    a. Daily
    b. Weekly
    c. Occasionally

9. On a typical occasion when you drank caffeinated beverages since your last study visit, how many drinks did you consume (if you drank some every day, answer for a typical day)
    a. Caffeinated coffee: number of 8 ounce cups (about the size of a mug): __(0-10,More than 10)
    b. Caffeinated tea: number of 8 ounce cups (about the size of a mug) __(0-10,More than 10)
    c. Cans (12 oz) or bottles (16 oz) of soda with caffeine (for example, Coke, Pepsi, Dr. Pepper, Mountain Dew. Count diet and regular together): __(0-10,More than 10)
    d. Cans (12 oz) or bottles (16 oz) of energy drinks with caffeine (for example, Red Bull, Amp): __(0-10,More than 10)

Changes In Your Asthma (women with asthma only)

Now we would like to get some information about how your asthma has been since your last study visit:

1. During the past 30 days, on how many nights did symptoms of asthma make it difficult for you to stay asleep?
    a. None
    b. Less than every night
        i. How many: ____ nights (response should be <30 nights)
    c. Every night
    d. Don’t know

2. Since your last study visit, have you had an episode of asthma or an asthma attack?
    a. Yes
    b. No (SKIP TO Q6)
    c. Don’t know (SKIP TO Q6)

3. Since your last study visit, how many asthma episodes or attacks have you had? (If you don't know the exact number, just give us your best guess)
    a. ____episodes

4. How long did your MOST RECENT asthma episode or attack last?
    a. __ __ # of minutes
    b. __ __ # of hours
    c. __ __# of days
    d. Don't know/Not sure

5. Compared with other episodes or attacks, was this most recent attack shorter, longer, or about the same?
    a. Shorter
    b. Longer
    c. About the same
    d. The most recent attack was actually the first attack
    e. Don't know

Now we would like to ask you about times when you seek treatment for your asthma:

6. Since your last study visit, how many times did you see a doctor or other health professional for a routine checkup for your asthma?
    a. None
    b. 1 time
    c. >1 time
        i. How many: ______
    d. Don't know

7. An urgent care center treats people with illnesses or injuries that must be addressed immediately and cannot wait for a regular medical appointment. Since your last study visit, have you had to visit an emergency room at a hospital or urgent care center because of your asthma?
    a. Yes
    b. No (SKIP TO Q9)
    c. Don't know (SKIP TO Q9)

8. Since your last study visit, how many times did you visit an emergency room or urgent care center because of your asthma?
    a. 1 time
    b. >1 time
        i. How many: _____
    c. Don't know

9. Since your last study visit, other than urgent care, how many times did you see a doctor or other health professional because of worsening asthma symptoms or for an asthma episode or attack?
    a. None
    b. 1 time
    c. >1 time
        i. How many: ______
    d. Don't know

10. Since your last study visit, have you had to stay overnight in a hospital because of your asthma? Do not include an overnight stay in the emergency room.
    a. Yes
    b. No (SKIP TO NEXT SECTION)
    c. Don't know (SKIP TO NEXT SECTION)

11. Since your last study visit, how many different times did you stay in any hospital overnight or longer because of your asthma? (If you do not know exactly how many, just give us your best guess)
    a. _____ (response should be >0)

Changes To Physical Activity

1. SINCE your last study visit, when you are NOT at work, how much time do you usually spend:

		None	Less than ½ hour per day	½ to almost 1 hour per day	1 to almost 2 hours per day	2 to almost 3 hours per day	3 or more hours per day
a.	Preparing meals (cook, set table, wash dishes)	①	②	③	④	⑤	⑥
b.	Dressing, bathing, feeding children while you are sitting	①	②	③	④	⑤	⑥
c.	Dressing, bathing, feeding children while you are standing	①	②	③	④	⑤	⑥
d.	Playing with children while you are sitting or standing	①	②	③	④	⑤	⑥
e.	Playing with children while you are walking or running	①	②	③	④	⑤	⑥
f.	Carrying children	①	②	③	④	⑤	⑥
g.	Taking care of an older adult	①	②	③	④	⑤	⑥
h.	Sitting and using a computer or tablet or writing, while not at work	①	②	③	④	⑤	⑥
i.	Sitting and playing a video game while not at work	①	②	③	④	⑤	⑥

j.	Playing with pets	①	②	③	④	⑤	⑥
k.	Light cleaning (make beds, laundry, iron, put things away)	①	②	③	④	⑤	⑥
l.	Shopping (for food, clothes, or other items)	①	②	③	④	⑤	⑥
m.	Heavier cleaning (vacuum, mop, sweep, wash windows)	①	②	③	④	⑤	⑥
n.	Mowing lawn while on a riding mower	①	②	③	④	⑤	⑥
o.	Mowing lawn while using a walking mower, raking, gardening	①	②	③	④	⑤	⑥
p.	Walking slowly to go places (such as to the bus, work, visiting) *Not for fun or exercise*	①	②	③	④	⑤	⑥
q.	Walking more quickly to go places (such as to the bus, work, visiting) *Not for fun or exercise*	①	②	③	④	⑤	⑥
r.	Driving or riding in a car or bus	①	②	③	④	⑤	⑥
s.	Walking slowly for fun or exercise	①	②	③	④	⑤	⑥
t.	Walking more quickly for fun or exercise	①	②	③	④	⑤	⑥
u.	Walking quickly up hills for fun or exercise	①	②	③	④	⑤	⑥
v.	Jogging	①	②	③	④	⑤	⑥
w.	Prenatal exercise class	①	②	③	④	⑤	⑥
x.	Swimming	①	②	③	④	⑤	⑥
y.	Dancing	①	②	③	④	⑤	⑥
z.	Doing other things for fun or exercise? Please tell us what they are (or leave this blank if you	①	②	③	④	⑤	⑥

	have none to report). __ __ __						
aa.	Doing other things for fun or exercise? Please tell us up to three activities (or leave this blank if you have none to report).<br>i__________<br>ii___________<br>iii__________	①	②	③	④	⑤	⑥

2. SINCE your last study visit, how much time do you usually spend:

		None	Less than ½ hour per day	½ to almost 2 hours per day	2 to almost 4 hours per day	4 to almost 6 hours per day	6 or more hours per day
a.	Watching TV or a video	①	②	③	④	⑤	⑥
b.	Sitting and reading, talking or on the phone, **while <u>not</u> at work**	①	②	③	④	⑤	⑥
c.	Sitting **at work** or in class (If you were not working, please record "None")	①	②	③	④	⑤	⑥
d.	Standing or slowly walking **at work** while carrying things (If you were not working, please record "None")	①	②	③	④	⑤	⑥
e.	Standing or <u>slowly</u> walking **at work** <u>not</u> carrying anything (If you were not working, please record "None")	①	②	③	④	⑤	⑥
f.	Walking <u>quickly</u> **at work** while <u>carrying</u> things (heavier than a 1 gallon milk jug) (If you were not working, please record "None")	①	②	③	④	⑤	⑥
g.	Walking <u>quickly</u> **at work** not <u>carrying</u> anything (If you were not working, please record "None")	①	②	③	④	⑤	⑥

Changes To Sleep

Now we would like to get some information about things that may have changed about your general sleep patterns since your last study visit:

1. How many hours of sleep did you usually get per night in the past two months:
   a. On weekdays or workdays? (4-10,>10) hours
   b. On weekends? (4-10,>10) hours

2. How many minutes did it usually take for you to fall asleep at bedtime? ____minutes

3. How many minutes of wake time (waking up in the middle of the night) did you have during a typical night's sleep? ____minutes

The next two questions below refer to the times you got in and out of bed to sleep, not including naps.

4. Not including naps, what time did you usually go to bed?
   a. On weekdays or workdays? ____ hh:mm AM/PM
   b. On weekends? ____ hh:mm AM/PM

5. Not including naps, what time did you usually wake up?
   a. On weekdays or workdays? ____ hh:mm AM/PM
   b. On weekends? ____ hh:mm AM/PM

6. During a usual week, how many times did you nap for 5 minutes or more?
   a. None
   b. 1 or 2 times
   c. 3 or more times

The following questions ask about your sleep habits. Please check one of the following for each of the questions. Pick the answer that best describes how often you experienced the situation.

1. In the past month

		**No**	**Yes, Less than once a week**	**Yes, 1 or 2 times a week**	**Yes, 3 or 4 times a week**	**Yes, 5 or more times a week**
a.	Did you have trouble falling asleep?	☐	☐	☐	☐	☐
b.	Did you wake up several times at night?	☐	☐	☐	☐	☐
c.	Did you wake up earlier than you planned to?	☐	☐	☐	☐	☐

d. Did you have trouble getting back to sleep after you woke up too early?	☐	☐	☐	☐	☐
e. Did you take sleeping pills to help you sleep?	☐	☐	☐	☐	☐

2. Overall, your typical night's sleep <u>during the last 4 weeks</u> was?
   a. Very sound or restful
   b. Sound or restful
   c. Average quality
   d. Restless
   e. Very restless

3. What position did you usually wake up in?

	**On my left side mostly**	**On my right side mostly**	**Both sides just as much**	**On my back mostly**	**On my front mostly**	**Just as much on my side as on my front or back**	**Sitting up/propped up**	**Don't remember /don't know**
a. During the last week?	☐	☐	☐	☐	☐	☐	☐	☐
b. Last night?	☐	☐	☐	☐	☐	☐	☐	☐

4. How often did you usually wake up during the night?
   a. On average, during the last week? ____times per night
   b. Last night? ___times per night

5. During the night, how often did you have to get out of bed (for example, to use the toilet)?
   a. On average, during the last week? ____times per night
   b. Last night? ___times per night

6. Based on your experience in the last 4 weeks, what is the chance that you would doze off or fall asleep (not just "feel tired") in the following situations? (Check one box for each situation. If you are never or rarely in the situation, please give your best guess for what would happen.)

	**No chance**	**Slight chance**	**Moderate chance**	**High chance**
a. Sitting and reading?	☐	☐	☐	☐

b.	Watching tv?	◻	◻	◻	◻
c.	Sitting inactive in a public place (such as a theater or meeting)?	◻	◻	◻	◻
d.	Riding as a passenger in a car for an hour without a break?	◻	◻	◻	◻
e.	Lying down to rest in the afternoon when circumstances permit?	◻	◻	◻	◻
f.	Sitting and talking to someone?	◻	◻	◻	◻
g.	Sitting quietly after a lunch without alcohol?	◻	◻	◻	◻
h.	In a car while stopped for a few minutes in traffic?	◻	◻	◻	◻
i.	While driving?	◻	◻	◻	◻

The next set of questions ask about snoring and sleep apnea in the last 4 weeks.

7. In the last 4 weeks, have you snored?
   a. Yes
   b. No (SKIP TO Q11)
   c. Don't know (SKIP TO Q11)

8. In the last 4 weeks, your snoring has been?
   a. Slightly louder than breathing
   b. As loud as talking
   c. Louder than talking
   d. Very loud - could be heard in adjacent rooms

9. In the last 4 weeks, how often have you snored?
   a. Nearly every day
   b. 3-4 times a week
   c. 1-2 times a week
   d. 1-2 times a month
   e. Nearly never

10. In the last 4 weeks, has your snoring ever bothered other people?
    a. Yes
    b. No
    c. Don't know

11. In the last 4 weeks, has anyone noticed that you quit breathing during your sleep?
    a. Nearly every day
    b. 3-4 times a week
    c. 1-2 times a week
    d. 1-2 times a month
    e. Nearly never
    f. Never

12. In the last 4 weeks, how often did you feel tired or fatigued after your sleep?

a. Nearly every day
b. 3-4 times a week
c. 1-2 times a week
d. 1-2 times a month
e. Nearly never
f. Never

13. During your waking time in the last 4 weeks, did you feel tired, fatigued or not up to par?
    a. Nearly every day
    b. 3-4 times a week
    c. 1-2 times a week
    d. 1-2 times a month
    e. Nearly never
    f. Never

14. In the last 4 weeks, did you ever nod off or fall asleep while driving a vehicle?
    a. Yes
    b. No (SKIP TO NEXT SECTION ON FATIGUE)
    c. Don't drive a vehicle (SKIP TO NEXT SECTION ON FATIGUE)

    14a. How often did this occur?
    a. Nearly every day
    b. 3-4 times a week
    c. 1-2 times a week
    d. 1-2 times a month
    e. Nearly never

Now we would like to ask you some questions about fatigue since your last study visit. Fatigue is when you feel weary, tired, or have a lack of energy.

For each of the following questions, indicate the number that most closely indicates how you have been feeling <u>during the past week</u>.

1. To what degree have you experienced fatigue?

☐1 ☐2 ☐3 ☐4 ☐5 ☐6 ☐7 ☐8 ☐9 ☐10

Not at all **A great deal**

If no fatigue (answer choice 1), skip to next section

2. How severe is the fatigue which you have been experiencing?

☐1 ☐2 ☐3 ☐4 ☐5 ☐6 ☐7 ☐8 ☐9 ☐10

Mild **Severe**

3. To what degree has fatigue caused you distress?

☐1 ☐2 ☐3 ☐4 ☐5 ☐6 ☐7 ☐8 ☐9 ☐10

No distress **A great deal of distress**

In the past week, rate on a scale from 1-10 (1=Not at all, 10=A great deal) the degree fatigue has interfered with your ability to:

(NOTE: Check the box to the right of each activity if you don't do the activity)

Fatigue interfered with your ability to:

4. **Do household chores:** Don't do activity ☐

☐1 ☐2 ☐3 ☐4 ☐5 ☐6 ☐7 ☐8 ☐9 ☐10

Not at all **A great deal**

5. **Cook:** Don't do activity ☐

☐1 ☐2 ☐3 ☐4 ☐5 ☐6 ☐7 ☐8 ☐9 ☐10

Not at all **A great deal**

6. **Bathe or wash:** Don't do activity ☐

☐1 ☐2 ☐3 ☐4 ☐5 ☐6 ☐7 ☐8 ☐9 ☐10

Not at all **A great deal**

7. **Dress:** Don't' do activity ☐

☐1 ☐2 ☐3 ☐4 ☐5 ☐6 ☐7 ☐8 ☐9 ☐10

Not at all **A great deal**

8. **Work:** Don't do activity ☐

☐1 ☐2 ☐3 ☐4 ☐5 ☐6 ☐7 ☐8 ☐9 ☐10

Not at all **A great deal**

9. **Visit or socialize with friends or family**: Don't do activity ☐

☐1 ☐2 ☐3 ☐4 ☐5 ☐6 ☐7 ☐8 ☐9 ☐10

Not at all **A great deal**

10. **Engage in sexual activity:** Don't do activity ☐

☐1 ☐2 ☐3 ☐4 ☐5 ☐6 ☐7 ☐8 ☐9 ☐10

Not at all **A great deal**

11. **Engage in leisure and recreational activities**: Don't do activity ☐

☐1 ☐2 ☐3 ☐4 ☐5 ☐6 ☐7 ☐8 ☐9 ☐10

Not at all **A great deal**

12. **Shop and do errands**: Don't do activity ☐

☐1 ☐2 ☐3 ☐4 ☐5 ☐6 ☐7 ☐8 ☐9 ☐10

Not at all **A great deal**

13. **Walk:** Don't do activity ☐

☐1 ☐2 ☐3 ☐4 ☐5 ☐6 ☐7 ☐8 ☐9 ☐10

Not at all **A great deal**

14. **Exercise, other than walking:** Don't do activity ☐

☐1 ☐2 ☐3 ☐4 ☐5 ☐6 ☐7 ☐8 ☐9 ☐10

Not at all **A great deal**

15. Over the past week, how often have you been fatigued?
    a. Every day
    b. Most, but not all days
    c. Occasionally, but not most days
    d. Hardly any days

16. To what degree has your fatigue changed during the past week?
    a. Increased
    b. Fatigue has gone up and down
    c. Stayed the same
    d. Decreased

Health

Now we would like to ask you some questions about your emotions and how you've been feeling lately.There are no "right" or "wrong" answers to any of these questions.

For the following 20 items, please select the choice that best describes how you have felt ***over the past week***:

		Rarely (< 1 day) or never	Some or a little of the time (1-2 days)	Occasionally or a moderate amount of the time (3-4 days)	Most or all of the time (5-7 days)
1	I was bothered by things that don't usually bother me.	①	②	③	④
2	I did not feel like eating; my appetite was poor.	①	②	③	④
3	I felt that I could not shake off the blues even with the help from my family and friends.	①	②	③	④
4	I felt that I was not as good as other people.	①	②	③	④
5	I had trouble keeping my mind on what I was doing.	①	②	③	④
6	I felt depressed.	①	②	③	④
7	I felt that everything I did was an effort.	①	②	③	④
8	I felt hopeless about the future.	①	②	③	④
9	I thought my life had been a failure.	①	②	③	④
10	I felt fearful.	①	②	③	④
11	My sleep was restless.	①	②	③	④
12	I was unhappy.	①	②	③	④
13	I talked less than usual.	①	②	③	④
14	I felt lonely.	①	②	③	④
15	People were unfriendly.	①	②	③	④

16	I did not enjoy life.	①	②	③	④
17	I had crying spells.	①	②	③	④
18	I felt sad.	①	②	③	④
19	I felt that people disliked me.	①	②	③	④
20	I could not get “going”.	①	②	③	④

With this next set of questions we want to know about your level of anxiety *over the last 2 weeks*:

1. Over the last 2 weeks, how many days have you been nervous, anxious, or on edge?
   a. 0
   b. 1-2
   c. 3-4
   d. >4
   e. Don't know

2. Over the last 2 weeks, how many days have you not been able to stop or control worrying?
   a. 0
   b. 1-2
   c. 3-4
   d. >4
   e. Don't know

3. Over the last 2 weeks, how many days have you worried too much about different things?
   a. 0
   b. 1-2
   c. 3-4
   d. >4
   e. Don't know

4. Over the last 2 weeks, how many days have you had trouble relaxing?
   a. 0
   b. 1-2
   c. 3-4
   d. >4
   e. Don't know

5. Over the last 2 weeks, how many days have you been so restless that it was hard to sit still?
   a. 0
   b. 1-2
   c. 3-4
   d. >4
   e. Don't know

6. Over the last 2 weeks, how many days have you been easily annoyed or irritable?
   a. 0
   b. 1-2
   c. 3-4
   d. >4
   e. Don't know

7. Over the last 2 weeks, how many days have you felt afraid as if something awful might happen?

a. 0
b. 1-2
c. 3-4
d. >4
e. Don't know

Now we would like to know about stress in your life in the last month.

For each question, please check the answer that is most true for you. There are no "right" or "wrong" answers.

		Never (1)	Almost Never (2)	Some-times (3)	Fairly Often (4)	Often (5)
1	In the last month, how often have you been upset because of something that happened unexpectedly?	①	②	③	④	⑤
2	In the last month, how often have you felt that you were unable to control the important things in your life?	①	②	③	④	⑤
3	In the last month, how often have you felt nervous and “stressed’?	①	②	③	④	⑤
4	In the last month, how often have you dealt successfully with irritating life hassles?	①	②	③	④	⑤
5	In the last month, how often have you felt that things were going your way?	①	②	③	④	⑤
6	In the last month, how often have you found you could not cope with all the things that you had to do?	①	②	③	④	⑤
7	In the last month, how often have you been able to control irritations in your life?	①	②	③	④	⑤
8	In the last month, how often have you felt that you were on top of things?	①	②	③	④	⑤
9	In the last month, how often have you been angered because of things that happened that were outside of your control?	①	②	③	④	⑤

10	In the last month, how often have you felt difficulties were piling up so high that you could not overcome them?	①	②	③	④	⑤
