# Supplementary material for: Maternal body composition and gestational weight gain in relation to asthma control during pregnancy
Source: PLoS One. 2022 Apr 20;17(4):e0267122. doi: 10.1371/journal.pone.0267122 (PMC9020691; doi:10.1371/journal.pone.0267122)
Supplement: S3 File — (PDF) [file pone.0267122.s015.pdf]

Asthma Cohort Study
Daily Diary
Draft February 11, 2014

Today's Date (MM/DD/YYYY)	autofill
**Health**	
Did you experience any of the following symptoms yesterday?	
Wheezing	YES NO
Coughing	YES NO
Shortness of Breath	YES NO
Chest Tightness	YES NO
Chest Pain	YES NO
Fever	YES NO
Nausea or vomiting	YES NO
Runny nose	YES NO
Other problem you want to tell us about?	Write in? Drop down?
Did you miss school, work or normal daily activities because of your illness or symptoms?	YES NO
Did you take any prescription medications yesterday?	YES NO
If Yes, please enter the medication name in the text field:	Text autocomplete field using the FDA approved drug product list (http://www.accessdata.fda.gov/scripts/cder/drugsatfda/). Application will present the user with all medications entered in previous diaries unless they remove the med from their med list. User will be able to add new meds if current med is not yet captured in their med list.
Dose	Text autocomplete field using the FDA approved drug product list dosage information (http://www.accessdata.fda.gov/scripts/cder/drugsatfda/). Dose will be carried forward from previous diary entries but user can modify if needed.
Frequency per day	Codelist options 1-10, 10+. Frequency will be carried forward from previous diary entries but user can modify if needed.

Did you take any over the counter medications or supplements yesterday?	YES NO
If Yes, please enter the OTC medication or supplement name in the text field:	Text autocomplete field for prenatal vitamins and low dose aspirin, all other meds will be entered as free text. Application will present the user with all medications entered in previous diaries unless they remove the med from their med list. User will be able to add new meds if current med is not yet captured in their med list.
Dose	Text field, Dose will be carried forward from previous diary entries but user can modify if needed.
Frequency per day	Codelist options 1-10, 10+, Frequency will be carried forward from previous diary entries but user can modify if needed.
**Daily Activities**	
Please indicate the number of hours and minutes that you slept last night	(Drop down) 0-10, 10+ hours 0, 15, 30, 45 minutes
Did you wake due to difficulty breathing or coughing in the middle of the night?	YES NO
**If yes**, enter the number of times you woke up:	0, 1, 2, 3, 4 or more
Please estimate the number of hours and minutes that you spent sitting yesterday.	(Drop down) 0, 1-10, 10+ hours 0, 15, 30, 45 minutes
Did you exercise yesterday?	YES NO
**If yes,** enter the number of minutes you spent exercising indoors.	0, <30, 30-45, 46-60, >60
**If yes,** enter the number of minutes you spent exercising outdoors.	0, <30, 30-45, 46-60, >60
Please estimate the number of hours and minutes that	

you spent:	
Outdoors	(Drop down) 0, 1-10, 10+ hours 0, 15, 30, 45 minutes
In a vehicle or other form of transportation (commuting for example)	(Drop down) 0, 1-10, 10+ hours 0, 15, 30, 45 minutes
Did you smoke yesterday?	YES NO
**If yes**, enter the number of cigarettes smoked	(Drop down) 1, 2, 3, 4, 5, 10, 20, >20 AUTOFILL
Were you around people yesterday while they were smoking?	YES NO
Did you drink any caffeinated beverages yesterday (colas, coffee, tea, energy drinks, etc.)?	YES NO
What was your level of daily stress?	1=Not Stressful 2=A Little Stressful 3=Very Stressful
